# Blumgart Anastomosis After Pancreaticoduodenectomy. A Comprehensive Systematic Review, Meta-Analysis, and Meta-Regression

**DOI:** 10.1007/s00268-021-06039-x

**Published:** 2021-03-15

**Authors:** Claudio Ricci, Carlo Ingaldi, Laura Alberici, Nico Pagano, Cristina Mosconi, Giovanni Marasco, Francesco Minni, Riccardo Casadei

**Affiliations:** 1 Department of Internal Medicine and Surgery (DIMEC), Alma Mater Studiorum, , S.Orsola-Malpighi Hospital, University of Bologna, Policlinico S.Orsola-Malpighi Via Massarenti n.9, 40138 Bologna, Italy; 2 Division of Pancreatic Surgery, Azienda Ospedaliero-Universitaria Di Bologna, via Albertoni 15, Bologna, Italia; 3 Division of Gastroenterology, Azienda Ospedaliero-Universitaria Di Bologna, via Albertoni 15, Bologna, Italia; 4 Division of Radiology, Azienda Ospedaliero-Universitaria Di Bologna, via Albertoni 15, Bologna, Italia; 5 Division of Internal Medicine, Azienda Ospedaliero-Universitaria Di Bologna, via Albertoni 15, Bologna, Italia

## Abstract

**Background:**

The superiority of Blumgart anastomosis (BA) over non-BA duct to mucosa (non-BA DtoM) still remains under debate.

**Methods:**

We performed a systematic search of studies comparing BA to non-BA DtoM. The primary endpoint was CR-POPF. Postoperative morbidity and mortality, post-pancreatectomy hemorrhage (PPH), delayed gastric emptying (DGE), reoperation rate, and length of stay (LOS) were evaluated as secondary endpoints. The meta-analysis was carried out using random effect. The results were reported as odds ratio (OR), risk difference (RD), weighted mean difference (WMD), and number needed to treat (NNT).

**Results:**

Twelve papers involving 2368 patients: 1075 BA and 1193 non-BA DtoM were included. Regarding the primary endpoint, BA was superior to non-BA DtoM (RD = 0.10; 95% CI: −0.16 to −0.04; NNT = 9). The multivariate ORs' meta-analysis confirmed BA's protective role (OR 0.26; 95% CI: 0.09 to 0.79). BA was superior to DtoM regarding overall morbidity (RD = −0.10; 95% CI: −0.18 to −0.02; NNT = 25), PPH (RD = −0.03; 95% CI −0.06 to −0.01; NNT = 33), and LOS (− 4.2 days; −7.1 to −1.2 95% CI).

**Conclusion:**

BA seems to be superior to non-BA DtoM in avoiding CR-POPF.

**Supplementary Information:**

The online version contains supplementary material available at 10.1007/s00268-021-06039-x.

## Introduction

The pancreatic anastomosis after pancreaticoduodenectomy (PD) still remains the main challenge for the pancreatic surgeon [1]. In the last 30 years, the pancreatic surgeons constantly attempted the "optimal" technical solutions in remnant management, producing some evidence about the best choices [2]. Nevertheless, many surgeons continue to propose a technical variant of pancreatic anastomosis, searching for the best reconstruction after PD [3]. Recently, a relative "novel" type of duct to mucosa called "Blumgart anastomosis" (BA) [4] has gained popularity. Several retrospective [5–12] studies have empathized BA efficacy in avoiding clinically relevant pancreatic fistula (CR-POPF). However, two recent randomized studies [13, 14] and two retrospective studies with propensity score matching (PSM) adjustment [15, 16] reported conflicting results about the BA advantages compared to non-Blumgart duct to mucosa (non-BA DtoM). Two recent meta-analyses, both including only one of three low-risk studies, suggest an advantage of BA in terms of B/CPOPF [17, 18]. The different results could be explained only by considering the bias due to most of the studies' retrospective design. Thus, the present study aims to rerun the meta-analysis, including all low-risk studies available.

The primary aim is to compare the two types of duct to mucosa using a reliable, useful, and reproducible parameter of safety, such as the CR-POPF rate according to the 2016 ISGPF definition [19]. The secondary endpoints are the overall and specific postoperative complications rate, including the overall mortality and morbidity rate, the post-pancreatectomy hemorrhage (PPH), and the delayed gastric emptying (DGE) rates defined according to ISGPS definition [20, 21]. We also evaluated the reoperation rate and the length of stay (LOS). We also performed a meta-regression analysis weighing the confounding factors' role.

## Material and methods

The manuscript was organized following the recommendations of Preferred Reporting Items for Systematic Reviews and Meta-Analyses statement (PRISMA) [22].

### Eligibility criteria

Eligibility criteria were established using the "Participants, Intervention, Control, Outcomes, Study" (PICOS) approach: (1) the participants were patients having benign or malignant pancreatic head lesions; (2) the intervention was the open PD with reconstruction according to BA anastomosis; (3) the control arm was the PD with reconstruction according to non-BA DtoM anastomosis; (4) the primary outcome was the CR-POPF rate; and (5) prospective randomized controlled trials and non-randomized prospective or retrospective controlled studies. No other differences were considered about the technical variant of both BA and non-BA DtoM. Nonetheless, the impact of variants was considered in the adjustment of bias.

### Search strategy

No language, publication date, or status restrictions were used. A systematic literature search was done through Pubmed, Scopus, and the ISI Web of Science. The last research was performed on November 9, 2020. The search string used was the following: "((pancreatojejunostomy OR pancreaticojejunostomy OR Blumgart anastomosis OR ((pancreas OR pancreatic) AND (anastomosis OR anastomoses OR anastomose))) AND (surgery OR resection OR pancreatoduodenectomy OR pancreaticoduodenectomy OR Whipple OR PPPD OR technique OR reconstruction))." We used all related articles to enlarge the systematic search, and the references of included studies were examined. The systematic search results were managed with Thomson Reuters Endnote version X7.

### Study selection, inclusion and exclusion criteria

A PRISMA flowchart was plotted to report the conclusions obtained by the authors. The studies were de-duplicated, and subsequently, they were screened according to the title and abstract to remove the records not relevant for this study's aim. Thus, the eligibility was evaluated in the remaining full-text articles using the following inclusion and exclusion criteria. Briefly, the inclusion criteria were: (1) comparative, randomized or non-randomized, design; (2) as intervention arm, any BA technique after PD; as control arm, any non-BA DtoM after PD; (3) the presence of POPF rate in both arms according to the 2016 ISGPF definition [19] or according to 2005 ISGPF definition but reporting B and C grade [23]; and (4) humans only clinical study. We defined BA as any anastomosis using trans-pancreatic sutures in the external layer and duct to mucosa in the inner layer. In the comparator arm, we included any duct to mucosa using other external layer methods. In Supplementary Table 1, all technical variants were described.

The following criteria were used to exclude studies: (1) studies that did not report original data or reviews or meta-analyses; (2) studies with data reported in untractable form; (3) studies that did not report data about CR-POPF; and (4) others type of anastomosis such as pancreaticogastrostomy in the control arm. The selection was independently performed in a blinded manner using a standardized form by two different authors (C.I. and L.A.). All eligible papers were reviewed in full-text form, and all the studies that met all the inclusion criteria without exclusion were selected for the analysis. Any disagreement was resolved after a collegial discussion between the reviewers and the senior author (R.C.).

### Data collection process and data item

Two reviewers (C.I. and C.M.) performed the data extraction using a preformed excel spreadsheet^©^. The following information was extracted to define each study's characteristics: authors, affiliation and country, year of publication, type of design (randomized or non-randomized), the technique of BA and non-BA DtoM anastomosis, the sample size of each arm, and the outcomes reported. On the contrary, the following data were extracted for the analysis: clinically relevant POPF, mortality, morbidity, B and C PPH, B and C DGE, reoperation rates, and LOS. All the disagreements were solved with a discussion between the reviewers and the last author (R.C.).

### Risk of bias in individual studies, summary measures, and synthesis of results

The studies' qualitative assessment was carried out based on the methodological index for non-randomized studies (MINORS) [24]. All categorical variables were reported as frequencies and percentages, while the continuous variables were described as means and standard deviations. A dedicated statistical algorithm was used to calculate the mean and standard deviation in studies that presented medians and interquartile ranges [25, 26]. The results were reported, for dichotomous variables, as risk difference (RD) with a 95% confidence interval (95% CI). The RD was used instead of the odds ratio (OR) to avoid an increase in the target event's rate. The study's primary endpoint was a relatively rare event, and some cells with zero value were expected [27]. Moreover, these results were reported as number needed to treat (NNT) calculated using the following formula $NNT=\frac{1}{ARR}=\frac{1}{RD}$ in which ARR represents the absolute risk reduction, namely the RD of the target event. The NNT value means the number of subjects who would have to undergo the BA rather than the non-BA DtoM to prevent one more event (e.g., CR-POPF). When RD with 95% CI assumes a positive value, the NNT was not calculated because the BA anastomosis did provide any clinical advantage. The primary endpoint were also analyzed using the ORs of multivariate models predicting CR-POPF when reported. Thus, this result was calculated using directly the ORs derived from each study. In this case, the results we reported only overall OR and NNT were not calculated. For continuous values such as LOS, we reported the weighted mean difference (WMD) with a 95% CI. The meta-analysis was carried out in line with recommendations from the Cochrane Collaboration and Meta-analysis of Observational Studies in Epidemiology guidelines [28, 29], and the Mantel–Haenszel random-effects model was used to calculate effect sizes [30].

### Risk of bias across studies and additional analyses

The risk of bias across included studies was tested, measuring both the "between-study heterogeneity" and publication bias. The heterogeneity was measured by testing both I^2^ and Cochran's Q statistics [31]. The *I*^2^ value reports the percentage of variation across the included studies related to heterogeneity rather than sampling error. The heterogeneity was interpreted as follows: If *I*^2^ was < 50%, the risk of "between-study" heterogeneity was considered low–moderate, and if I^2^ was ≥ 50%, it was judged high. The meta-regression analysis was carried out when the heterogeneity is high, and the result is statistically relevant [32]. The meta-regression was not planned for the primary endpoint based on ORs derived from multivariate analysis because it judged, in itself, not a risk. The meta-regression was based on the use of maximum residual likelihood (REML) [33, 34]. We calculated the distribution of confounding covariates among each arm in the first step, reporting the results as RR or WMD with an entire 95% CI. We also calculated the frequency of confounding variables that changed among the studies, such as MINORS score, type of BA, or non-BA DtoM. In the second step, β value with standard error (SE) and R^2^ was reported. The *β* value ± SE was related to the change in the RD unit of the target event: a positive *β* value means that the covariate increased rate generates a positive RD modification. The *R*^2^ measured the quote, in percentage, of the heterogeneity explained by the variable. A two-tailed *P* value < 0.05 was judged significant. The *P* values were also recalculated using Monte Carlo permutation to obtain robust results [35]. The Begg and the Egger test [36] was used to exploring the presence of the publication bias, and a *P*-value < 0.05 indicated a non-negligible "small-study effect." The statistical analysis was carried out using dedicated packages for STATA v14®.

## Results

### Studies selection, characteristics, and risk of bias within the studies

**Fig. 1:**
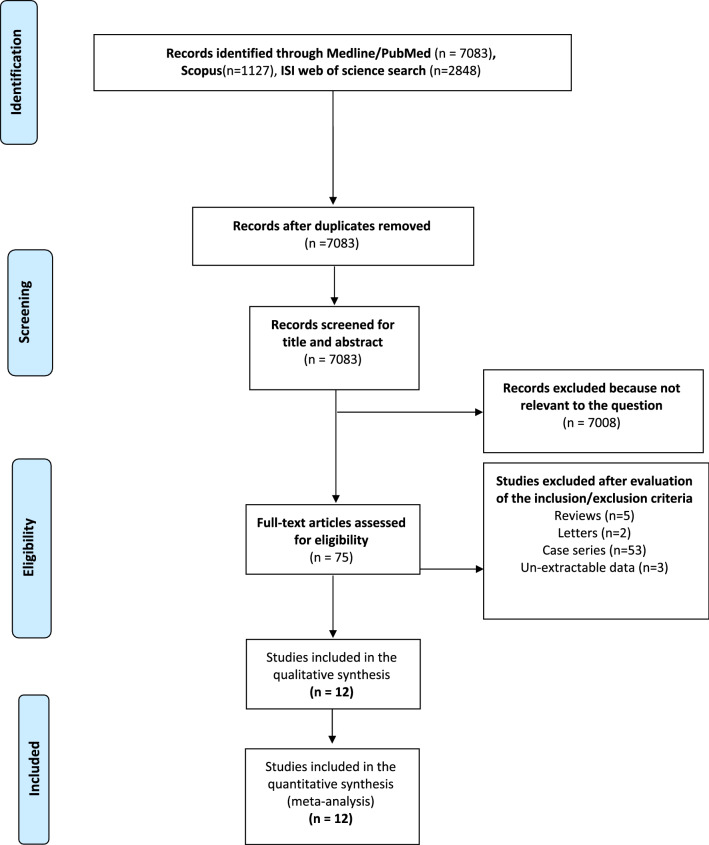
Selection process

The results of the systematic search of the literature following the PRISMA statement are reported in Fig. 1. The search identified 10,098 papers: 7083 from the Medline/PubMed database, 1127 from the ISI Web of Science, and 2848 from Scopus. Six thousand one hundred twenty-three titles were leftover after de-duplication. Of these, 7008 were excluded from evaluating the title and abstract because they were not pertinent to our study field. Seventy-four were reviewed in full-text form, and of these, 63 were excluded because: 53 were case series; 5 were reviews without original data; 2 were letters to the editor; and 3 contained unextractable data. Finally, twelve studies [5–16] were available for quality assessment and quantitative synthesis. There was 100% agreement between the two reviewers. The characteristics of the studies selected are summarized in Table 1. All the studies were published in the twenty-first century and only four [5, 14–16] in a Western country (33.3%). The majority of studies (8 out of 12, 66.7%) did not have a randomized design nor propensity score matching adjustment (PSM). In most studies (9/12, 75%), the original technique described by Blumgart [5] was modified. The main modification was the reduction of interrupted trans-pancreatic sutures from 4 or 6 to 2 or 3. There were 6 studies (50%) reporting non-BA DtoM according to the Cattel–Warren technique [37] and 6 (50%) reporting Kakita one in the control group [38]. A total of 2368 patients were recorded: 1075 (45.4%) undergoing BA and 1193 (54.6%) non-BA DtoM. A detailed description of all surgical techniques is reported in Supplementary Table 1. The other potential confounding variables, varying among the two arms, are summarized in Supplementary Table 2. Briefly, gender and age were extractable for all studies except Halloran et al. [14]; type of lesion and pancreatic texture were frequently available (10 and 9 studies, respectively), while using somatostatin analogs and Wirsung's dilatation rarely could be inferred (5 and 6 studies). The use of pancreatic stent was extremely variable within each study and not extractable. The two groups were well matched for gender, type of tumor (PDAC/CP vs. others), "soft pancreas," and not dilated Wirsung rate. The non-BA DtoM population was younger than BA one (WMD −1.59 years; *P* = 0.004), while the use of somatostatin analogs was more frequent in the BA group than non-BA DtoM one (RR 1.27; *P* = 0.002). Only in five studies [5, 7, 11, 13, 15], the surgeon's expertise was specified. The quality of included studies was reasonably good, with a median of 15 points (13–23) of MINORS score.

**Table 1:** Characteristics of the studies included

Authors	Affiliation/hospital	Year	Study design	Type of comparison	Sample size		Outcomes reported	Surgeon’s expertise	MINORS score
					BA	Non-BA DtoM			
Kleespies et al. [5]	Department of Surgery, Klinikum Grosshadern, University of Munich, Germany	2009	Retrospective without PSM	c-BA vs. CW-DtoM	92	90	CR-POPF, mortality, morbidity, PPH, reoperation, LOS,	Senior	14/24
Fujii et al. [6]	Department of Gastroenterological Surgery (Surgery II), University Graduate School of Medicine, Nagoya, Japan	2014	Retrospective without PSM	m-BA vs. Ka-DtoM	120	120	CR-POPF*, mortality, morbidity, DGE, reoperation, LOS	Not reported	14/24
Oda et al. [7]	Department of Surgery, Clinical Sciences, University of Tsukuba, Japan	2015	Retrospective without PSM	m-BA vs. Ka-DtoM	78	78	CR-POPF, mortality, morbidity, PPH, reoperation, LOS	Senior/junior	14/24
Kawakatsu et al. [8]	Department of Gastroenterological Surgery, Cancer Institute Hospital, Japanese Foundation for Cancer Research. Tokyo, Japan	2018	Retrospective without PSM	m-BA vs. Ka-DtoM	110	176	CR-POPF*, mortality, morbidity, reoperation, LOS	Not reported	15/24
Kojima et al. [9]	Department of Surgery, Okayama Saiseikai General Hospital, Okayama, Japan	2018	Retrospective without PSM	m-BA vs. CW-DtoM	101	103	CR-POPF*, morbidity, PPH, DGE, LOS	Not reported	15/24
Lee et al. [10]	Department of Surgery, Jesus Hospital Jeonju, Korea	2018	Retrospective without PSM	m-BA vs. CW-DtoM	43	44	CR-POPF, mortality, morbidity, PPH, DGE, LOS	Not reported	13/24
Hirono et al. [13]	Second Department of Surgery, Wakayama Medical University, School of Medicine, Wakayama, Japan	2019	RCT	m-BA vs. Ka-DtoM	107	103	CR-POPF, mortality, morbidity, PPH, reoperation, LOS	Senior	23/24
Li et al. [11]	Department of Hepato-biliary Surgery, Tianjin’s Clinical Research Center for Cancer, China	2019	Retrospective without PSM	m-BA vs. CW-DtoM c-BA vs. CW-DtoM	73 75	81	CR-POPF*, mortality, PPH, DGE, reoperation, LOS	Senior	16/24
Satoi et al. [12]	Department of Surgery, Kansai Medical University, Osaka, Japan	2019	Retrospective without PSM	m-BA vs. Ka-DtoM	118	128	CR-POPF*, mortality, morbidity, PPH, DGE, reoperation, LOS	Not reported	15/24
Casadei et al. [15]	Department of pancreatic surgery, University of Bologna, Bologna, Italy	2020	Retrospective with PSM	m-BA vs. CW-DtoM	37	37	CR-POPF*, mortality, morbidity, PPH, DGE, reoperation, LOS	Senior	19/24
Halloran et al. [14]	Pancreas Biomedical Research Unit and Clinical Directorate of General Surgery, University of Liverpool, Liverpool, UK	2020	RCT	m-BA vs. C_W-DtoM	112	124	CR-POPF*, mortality, morbidity, LOS, QoL, entry to chemo	Not reported	23/24
Menonna et al. [16]	Division of General and Transplant Surgery, Azienda Ospedaliero Universitaria Pisana. University of Pisa, Italy	2020	Retrospective with PSM	m-BA vs. CW-DtoM	109	109	CR-POPF, mortality, morbidity, PPH, DGE, reoperation, LOS	Not reported	19/24
Total					1175	1193			15/24*

*PSM* propensity score matching; *RCT* randomized controlled trial; *BA* Blumgart anastomosis; *c-BA* classic Blumgart anastomosis; m-BA = modified Blumgart anastomosis; non-BA DtoM = duct to mucosa anastomosis different from BA; CW-DtoM = Cattel–Warren duct to mucosa anastomosis; Ka-DtoM = Cattel–Warren duct to mucosa anastomosis; CR-POPF = clinically relevant postoperative pancreatic fistula according to 2017 International Study group of pancreatic fistula (ISGPF) definition; * = the data were calculated using both crude rate of CR-POPF and odds ratio of CR-POPF derived from multivariate analysis. *PPH* post-pancreatectomy hemorrhage; *DGE*   delayed gastric emptying; *LOS* length of stay

### Results of individual studies and synthesis of the results

**Fig. 2:**
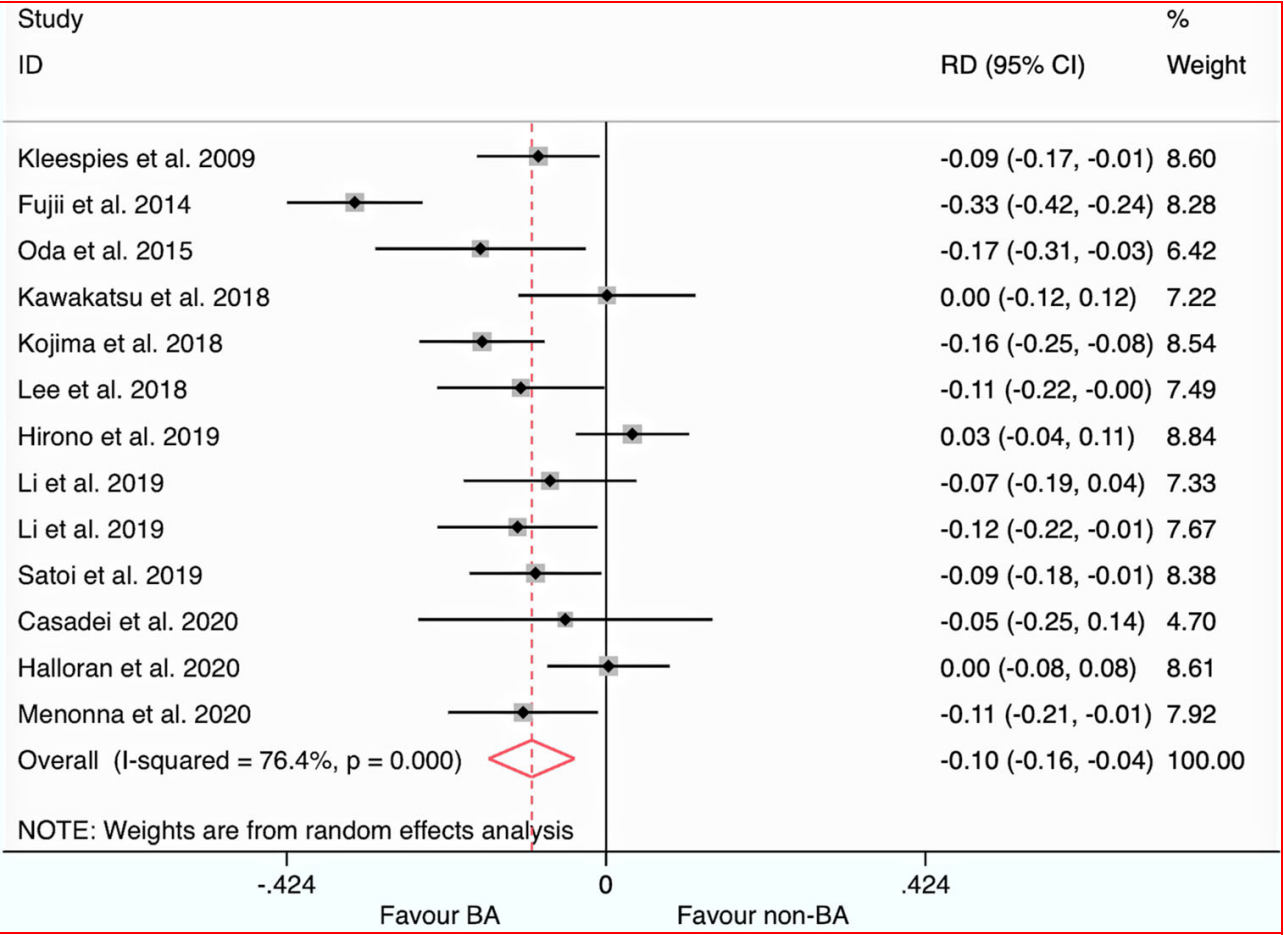
Forest plot for the primary endpoint. *Legend* ID = identification of the study (name of first authors and year of publication); *RD* risk difference; 95% CI: 95% confidence interval; *BA*   Blumgart anastomosis; non-BA = non-Blumgart duct to the mucosa. *I*^2^: heterogeneity; gray square: risk difference of each study; size of square: the weight of each study; solid black line: the 95% confidence interval; maroon diamond: the cumulative risk difference; dotted maroon line: the overall effect

The funnel plot of each endpoint is plotted in Supplementary Fig. 1 Panel a–g. The meta-analytic data are summarized in Fig. 2 (primary endpoint) and Supplementary Fig. 2 Panel a–f (secondary endpoints) and synthesized in Table 2. The "between-study" heterogeneity and publication bias are also reported in Table 2.

**Table 2:** Meta-analysis of all outcomes

Outcomes of interest	No. of studies	Event rate (%) o weighted mean (SD)		RD, OR, or WMD (95% CI)	NNT	P-value	Heterogeneity P-value of C-Q, I^2^ (%)	P-value for reporting bias ^	
		BA arm	non-BA DtoM arm					Egger	Begg
*Primary endpoint*									
CR-POPF	12	146/1175 (12.4)	282/1193 (23.6)	−0.10 (−0.16 to −0.04)	9 (7 to 12)	0.001	< 0.001; 51	0.030	0.044
CR-POPF based on multivariate ORs	5	§	§	0.26 (0.09 to 0.79)	§	0.017	< 0.001; 75.1	0.149	0.221
*Secondary endpoints*									
Mortality	11	12/962 (1.2)	26/966 (2.7)	−0.01 (-0.02 to 0.01)	70 (*)	0.258	0.093; 38.4	0.410	0.462
Overall morbidity	11	360/915 (39.4)	381/879 (43.3)	−0.10 (−0.18 to −0.02)	25 (11 to 180)	< 0.001	< 0.001; 71.5	0.656	0.602
PPH grade B and C°	11	24/953 (2.5)	50/893 (5.6)	−0.03 (−0.06 to −0.01)	33 (20 to 78)	0.022	< 0.001; 73	0.953	1.000
DGE grade B and C°	8	79/676 (11.7)	65/622 (10.4)	−0.01 (−0.04 to 0.04)	81** (*)	0.987	0.029; 55.1	0.121	0.063
Reoperation	11	32/919 (3.5)	43/922 (4.7)	−0.01 (−0.02 to 0.01)	85 (*)	0.429	0.298; 15.8	0.501	0.858
LOS (days)	11	20.2 (11.3)	24.7 (14.5)	−4.17 (−7.13 to −1.21)	§	0.006	< 0.001;84.2	§	§

*BA* Blumgart anastomosis; non-BA DtoM = duct to mucosa anastomosis different from BA;* SD* standard deviation; *RD* risk difference; *OR* odds ratio; *WMD* weighted mean difference; *NNT* number needed to treat; C-Q = Cochran’s test; *I*^2^ = Higgins test; ^ = A reporting bias non-negligible is considered for *P* values < 0.10; CR-POPF = clinically relevant postoperative pancreatic fistula; *PPH*  post-pancreatectomy hemorrhage according ISGPS classification; DGE = delayed gastric empting according ISGPS classification; *LOS*  length of stay; * = confidence interval not computable because the treatment results both harmful and helpful; § = not applicable; ** = the treatment results harmful

### Primary endpoint

**Fig. 3:**
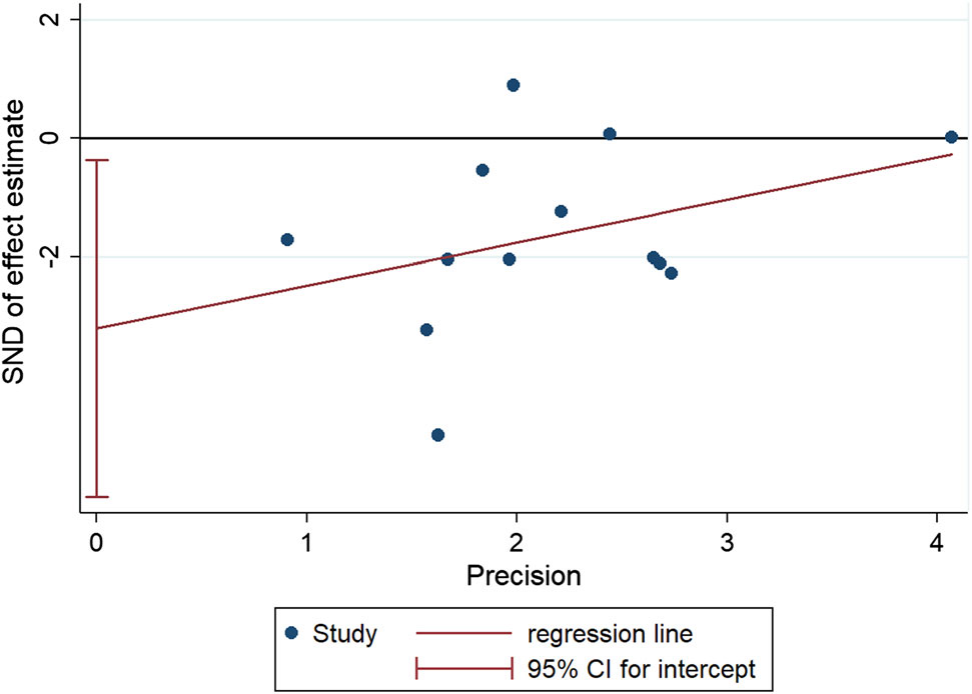
Regression line of Egger test for publication bias assessment. *Legend* vertical axis: the measure of the effect divided by the standard error (SND); horizontal axis: the precision of each study (1/standard error); red line: regression line; blue circle: each included study; vertical red line: the intercept and its 95% confidence interval

The risk of CR-POPF, calculated by extracting each event rate, was significantly lower in the BA arm than in the non-BA DtoM one: The RD was -0.10 (95% CI: −0.16 to −0.04; *P* = 0.002). The NNT was 9 (every ten patients managed with BA instead of non-BA DtoM, one CR-POPF more could be prevented). The 95% CI indicated that clinical advantage could be more significant in the best scenario (NNT = 7) or more minor in the worst one (NNT = 12). The results did not change rerunning the analysis, using the ORs derived from multivariate models of each study: The odds that CR-POPF was lower in the BA group than in non-BA DtoM one is statistically significant and clinically relevant (OR 0.26; 95% CI: 0.09–0.70; *P* = 0.017). The heterogeneity of the primary endpoint was not negligible (51% and 75.1%). The "small-study effect" was discovered for RD values with Begg (*P* = 0.030) and Egger tests (*P* = 0.044). The regression line, plotted in Fig. 3, suggested the absence in the literature of studies with a small sample size and not favorable for the intervention arm (BA).

### Secondary endpoints

No significant differences were found between the two techniques about mortality, B and C DGE, and reoperation rate. Overall morbidity rate was significantly lower than BA with non-BA DtoM (RD = −0.10; 95% CI: −0.18 to −0.02; *P* = 0.007) with an NNT of 25. The B and C PPH rate was significantly lower in the BA group than the non-BA DtoM one (RD = −0.03; 95% CI −0.06 to −0.01; *P* = 0.022). However, the clinical relevance of this statistical difference was very small (NNT = 33). The LOS was significantly reduced in patients who received a BA than those undergoing non-BA DtoM (WMD =−4.2 days; −7.1 to −1.2 95% CI; *P* = 0.006). Among the secondary endpoints, overall morbidity, PPH, and LOS have a not negligible heterogeneity, while none of these results were affected by publication bias.

### Meta-regression analysis

Meta-regression analysis for CR-POPF is reported in Table 3. The RD decreased when the included studies' quality increased (−0.02 ± 0.01; *P* = 0.028 ± 0.001). The remaining confounding parameters varying (gender, age, type of lesion (PDAC/CP), soft pancreas, use of somatostatin analogs, Wirsung not dilated type of BA, type of non-BA DtoM, and country) did not explain the heterogeneity. Meta-regression analysis for CR morbidity, PPH, DGE, and hospital stay is detailed in supplementary Tables 3–6.

**Table 3:** Results of univariate meta-regression analysis for CR-POPF

Covariates	Number of studies	Beta coefficient ± SE	Adjusted *R*^2^ (%)	*P*-value	*P*-value ± SE after Monte Carlo permutation
Study design (retrospective vs. PSM/RCT)	12	−0.10 ± 0.05	−28	0.073	0.073 ± 0.008
Male gender, RR	11	−0.01 ± 0.27	−12	0.948	0.950 ± 0.007
Age (years), WMD	11	0.01 ± 0.02	8	0.262	0.277 ± 0.014
PDAC or CP, RR	8	0.15 ± 0.17	−4	0.409	0.467 ± 0.016
“Soft pancreas,” RR	8	0.21 ± 0.27	−3	0.470	0.447 ± 0.016
Use of somatostatine analogues, RR	5	−0.32 ± 0.32	1	0.395	0.432 ± 0.016
Wirsung not dilated, RR	6	−0.39 ± 0.45	−9	0.433	0.433 ± 0.016
MINORS score	12	−0.02 ± 0.01	46	0.022	0.028 ± 0.001
Type of BA (c-BA vs. m-BA)	12	0.03 ± 0.06	−8	0.591	0.614 ± 0.015
Type of DtoM (CW-DtoM vs. Ka-DtoM)	12	−0.08 ± 0.06	10	0.212	0.206 ± 0.013
Study origin (Western vs. Eastern)	11	−0.06 ± 0.07	0	0.334	0.303 ± 0.015

*SE* standard error; BA = Blumgart anastomosis; CW-DtoM = Cattel–Warren duct to mucosa anastomosis; Ka-DtoM = Kakita duct to mucosa anastomosis; *PDAC* pancreatic ductal adenocarcinoma; *CP* chronic pancreatitis; *RR* risk ratio; *WMD* weighted mean difference; *R*^2^ = relative reduction in between-study variance: The value indicates the proportion of between-study variance explained by covariate; *RR* risk ratio; *MD* mean difference; *BMI* body mass index; *PDAC* pancreatic ductal adenocarcinoma; *CP* chronic pancreatitis; *PP* pylorus preserving pancreaticoduodenectomy; * = insufficient observation to perform Monte Carlo permutation

## Discussion

Our study confirmed that BA seems to reduce the risk of CR-POPF significantly when compared with non-BA DtoM (RD–11%; *P* < 0.001). The reduction seems to be clinically relevant with an NNT near nine, which means that one CR-POPF can be avoided every ten patients treated with BA instead of DtoM. However, this result should be interpreted with caution because the meta-regression analysis showed that RD's magnitude could be reduced in randomized and non-randomized well-designed studies.

Thus, several phenomena CR-POPF-related such as the overall complication rate (−10%, *P* = 0.007; NNT = 25), PPH (−3%; 0.023; NNT = 33), and LOS (−4 days; *P* = 0.005) were significantly influenced by BA. All these results did not surprise because it is well known that CR-POPF is the main reason for a complicated postoperative course, B/C PPH appearance, and consequently a long postoperative stay [39, 40]. No other statistical and clinical advantages were observed about mortality rate, DGE, and reoperation rate. These results were obtained with the largest meta-analysis available in the literature, to our knowledge.

Moreover, our study is the first meta-analysis that included both RCTs available. Indeed, we included 11 comparative studies (2 RCTs, 2 retrospectives with PSM, and eight non-randomized comparative), recently published (2009–2020) overall sample size of 2368 patients. This datum suggested, in itself, that the interest of pancreatic surgeons around the BA is high even if it is well known that other factors could influence the risk of pancreatic fistulae, such as the type of tumors or the characteristics of pancreatic remnant. Nonetheless, the BA popularity is probably due to the postoperative results and the simplicity of execution: In Fujii et al. [6] variant, only three trans-pancreatic sutures are needed for the external layer. Besides, this anastomosis seems to put together, for the first time, two principles dear to all pancreatic surgeons: On the one side, BA permits to perform a duct to mucosa anastomosis, minimizing the opening of jejunum, and at the same time, the pancreatic remnant was invaginated by a seromuscular jejunal loop.

Indeed, the external layer was made using trans-parenchymal U sutures tied on the jejunal loop, and this technical aspect was the main difference with other types of non-BA DtoM. The invagination with U sutures could reduce the severity of fistulas, minimizing the duct to mucosa failure. Moreover, the invagination with U sutures could reduce the effect of other phenomena related to the severity of POPF, such as PPH [40] or the high number of side branches in the soft pancreas [41, 42].

This study had some limitations, and the results of our meta-analysis had been interpreted with prudence for several reasons. The main limitation was that most included studies (72.7%) have a retrospective design without randomization or PSM adjustment. The risk of selection bias in favor of the BA arm could be not negligible. Moreover, a significant publication bias is present and easily interpreted: Small "negative" studies, namely in which BA failed, could remain unpublished. The meta-regression analysis confirmed this limitation, suggesting that RD could be inferior in well-designed studies such as RCTs or retrospective PSM-adjusted that have similar validity to the randomized studies [43–45]. Thus, it should be noted that the advantage of BA could be inferior in clinical practice. Second, most studies (66.7%) were carried out in Eastern countries, weakening the reproducibility of the results in Western ones for the differences in populations (BMI or fatty infiltration of the pancreatic remnant). Tough, this should not be the first time that a "new" pancreatic anastomosis has the best results in Eastern countries [46] but failed when used in Western ones [47, 48]. Third, as well demonstrated, the true test bed for a pancreatic anastomosis was the "soft pancreatic remnant." [49, 50] Indeed, BA safety evaluation could be conducted only in the "at-risk pancreas" rather than in an entire cohort of patients who underwent PD. Fourth, the expertise of surgeons was rarely reported, and this factor could influence the CR-POPF rate. Nonetheless, the affiliation seems to guarantee that all studies were conducted in high-volume pancreatic centers. Fifth, the power of meta-regression could be limited by lacking some data such as octreotide use or pancreatic remnant stiffness. Sixth, our study was limited to the open approach. The reason for this choice is to reduce the risk of bias because some difference in morbidity exists between the minimally invasive arms available [51]. Finally, the original technique described by Blumgart and Kleespies^5^ has undergone many changes, and each author introduced specific technical changes that could ameliorate the results. However, it is challenging to evaluate the impact of POPF's RD changes due to the high number of groups. Moreover, several differences in peri-operative management (for example, drain removal policy, mitigation strategies, enhanced principle adoption) could occur among the included studies, contributing to the results' variability.

Despite these limitations, our study clarifies that BA anastomoses could reduce the risk of CR-POPF. Consequently, phenomena POPF-related, such as overall morbidity rate or B/C PPH or long postoperative stay, could be decreased by adopting this reconstruction technique. However, these results need to be confirmed in further and well-structured studies conducted in Western countries, including only "at-risk pancreatic remnant" with randomized or non-randomized PSM-based design.

## Supplementary Information

Below is the link to the electronic supplementary material.

Supplementary file1 (DOCX 44 KB)

Supplementary file2 (DOCX 43069 KB)
