# Supplementary material for: Blumgart Anastomosis After Pancreaticoduodenectomy. A Comprehensive Systematic Review, Meta-Analysis, and Meta-Regression
Source: World J Surg. 2021 Mar 15;45(6):1929–39. doi: 10.1007/s00268-021-06039-x (PMC8093149; doi:10.1007/s00268-021-06039-x)
Supplement: Supplementary file 1 — Supplementary file1 (DOCX 44 KB) [file 268_2021_6039_MOESM1_ESM.docx]

**Supplementary Table 1. Techniques of pancreatic anastomosis used in included studies**

| Authors | Blumgart anastomoses | | Duct to mucosa anastomoses | |
| --- | --- | --- | --- | --- |
|  | External layer | Internal layer | External layer | Internal layer |
| Kleespies et al. ^5^ | Four trans-pancreatic U-sutures were placed straight through the pancreatic remnant about 1 cm distal from the cut end (absorbable, monofilament suture with MH1 needle).  Each of the sutures started at the ventral side of the gland, going from front to back straight through the pancreas.  This stitch was followed by a seromuscular stitch through the back wall of the jejunal loop, coming back through the pancreas from back to front, thereby approximating the jejunum to the dorsal face of the pancreatic remnant.  Each of the U-sutures was placed at a distance of 5–10 mm from the next. Two of each were placed cranial and two of each caudal to the pancreatic duct  Finally, after duct to mucosa anastomosis, the U-sutures were completed by placing both needles through the anterior portion of the jejunum, adapting the jejunum to the pancreas and tying the knots carefully at the ventral wall of the jejunum…” | Duct to mucosa anastomosis with interrupted monofilament absorbable sutures | “…Monofilament absorbable interrupted sutures were placed with an atraumatic needle, starting at the posterior surface of the pancreas.  The dorsal capsule of the pancreas was sutured to the seromuscular layer of the jejunum. After the completion of duct to mucosa anastomosis, the ventral part of the anastomosis was sutured in the same fashion…” | Duct to mucosa anastomosis with interrupted monofilament absorbable sutures..” |
| Fujii et al. ^6^ | Some modify of techniques reported by Kleepies et al.: only three penetrating sutures; non-absorbable monofilament; none of penetrating sutures were tied on the pancreas. | The pancreatic duct and the jejunal mucosa were joined in an end-to-side fashion using eight absorbable interrupted sutures. | After completing duct to mucosa anastomosis, the pancreatic parenchyma of the stump was approximated to the jejunal seromuscular layer with three or four non-absorbable interrupted penetrating sutures.  After ligation, of all interrupted duct-to-mucosa sutures, were placed through the seromuscular layer of the jejunum 5–7 mm lateral to the previous sutures | The pancreatic duct and the jejunal mucosa were joined in an end-to-side fashion using eight absorbable interrupted sutures. |
| Oda et al. ^7^ | Some modify of techniques reported by Kleepies et al.: only three penetrating sutures; absorbable monofilament; none of penetrating sutures were tied on the pancreas; semicircular seromuscular sutures. After completion of the MPD-jejunum anastomosis, needles of both horizontal mattress sutures were pierced through the anterior wall of the jejunum 1 cm from the pancreatic margin longitudinally to cover the seromuscular layer of the anterior semicircle. | The main pancreatic duct was anastomosed to the full thickness of the jejunum using 4 running adsorbable sutures; each takes responsibility for 90 degrees of the circumference. | Modified version of the technique reported by Fujii et al. : monofilament absorbable sutures. | The main pancreatic duct was anastomosed to the full thickness of the jejunum using 4 running adsorbable sutures, and each takes responsibility for 90 degrees of the circumference. |
| Kawakatsu et al.^8^ | See Oda et al. technique | Not specified | See Oda et al. technique | Not specified |
| Kojima et al. ^9^ | Some modify of techniques reported by Kleepies et al.: only three penetrating sutures; non-absorbable monofilament; none of penetrating sutures were tied on the pancreas. | Not specified | See Kleespies et al. technique | Not specified |
| Lee et al. ^10^ | See Kleespies et al. technique | Not specified | Some modify of technique reported by Kleespies et al.: non-absorbable sutures | Duct to mucosa with interrupted absorbable sutures non-monofilament |
| Hirono et al. ^13^ | See Fujii et al. technique but with absorbable sutures | Duct to mucosa anastomosis with interrupted monofilament absorbable sutures | The jejunal seromuscular layer was sutured to the pancreatic parenchyma of the stump in an interrupted penetrating fashion, using absorbable sutures. We usually used 4 trans-pancreatic jejunal seromuscular sutures based on the Kakita method | Duct to mucosa anastomosis with interrupted monofilament absorbable sutures |
| Li et al. ^11^ | See Fuji et al. technique for modified BA  See Kleespies et al. technique for classical BA | In modified BA, we superimposed the backwall sutures on each other, omitted the “duct to mucosa” anastomosis by fixing the stenting and tied the final knot on the jejunum surface | See Fujii et al. | See Fujii et al. |
| Satoi et al. .^12^ | See Fujii et al. | See Fujii et al. | See Fujii et al. | See Fujii et al. |
| Casadei et al. ^15^ | See Fuji et al. technique for modified BA | The pancreatic duct and the jejunal mucosa were joined in an end-to-side fashion using four to six absorbable interrupted sutures | Invagination pancreaticojejunostomy (PJ) includes an outer layer of posterior row of interrupted 3-4/0 polypropylene suture between the posterior surface of the pancreas remnant and the jejunum. Incision of the jejunal loop have to be performed slightly shorter than the pancreatic remnant and double (posterior and anterior) inner continuous suture with polydioxanone 5–6/0. Finally, outer layer of anterior row of interrupted 3–4/0 polypropylene suture between the anterior surface of the pancreas remnant and the jejunum | Interrupted 3–4/0 polypropylene suture between the posterior surface of the pancreas remnant and the jejunum; a duct-to- mucosa anastomosis with posterior and anterior interrupted 5–6/0 polydioxanone suture between the Wirsung duct and the small incision of the jejunal loop; and interrupted 3–4/0 polypropylene suture between the anterior surface of the pancreas remnant and the jejunum. |
| Halloran et al.^14^ | See Fuji et al. technique for modified BA  See Kleespies et al. technique for classical BA | See Fujii et al. | See Kleespies et al. technique | Not specified |
| Maronna et al. ^16^ | Some modify of techniques reported by Kleepies et al.: only two penetrating sutures; non-absorbable monofilament; none of penetrating sutures were tied on the pancreas but just once over the jejunum; A U-shaped suture was placed between the jejunum and the pancreatic capsule posterior to the pancreatic duct; a similar U-shaped suture was placed anterior to the pancreatic duct; 2 “half-purse string sutures” were placed at the corners of the  pancreatic anastomosis | See Fujii et al. | Some modify of techniques reported by Kleepies et al.:  non-absorbable monofilament | Some modify of techniques reported by Kleepies et al.:  non-absorbable monofilament |

**Supplementary Table 2. Other covariates potentially influencing the results of the meta-analysis. The results were reported as the proportion or weighted median difference among the two group**

| Covariates (BA vs. non-BA DtoM) | Number of studies | RR o WMD (95CI) | P-value |
| --- | --- | --- | --- |
| Male gender | 11 | 0.97 (0.91 to 1.01) | 0.404 |
| Age (years) | 12 | -1.59 (2.67 to -0.51) | 0.004 |
| PDAC and CP | 10 | 1.04 (0.93 to 1.15) | 0.521 |
| “Soft pancreas” | 9 | 0.93 (0.85 to 1.02) | 0.130 |
| Use of somatostatine analogues | 5 | 1.27 (1.09 to 1.48) | 0.002 |
| Wirsung not dilated | 6 | 0.95 (0.85 to 1.06) | 0.360 |

**Legend**: BA= Blumgart Anastomosis; non-BA DtoM= Duct to mucosa anastomosis different from BA; PDAC= Pancreatic ductal adenocarcinoma; CP= chronic pancreatitis; RR= risk ratio; WMD= Weighted Mean Difference; 95 CI= Confidence Interval at 95 %.

**Supplementary Table 3. Results of univariate meta-regression analysis for overall morbidity**

| Covariates | Number of studies | Beta coefficient ± SE | | Adjusted R^2^ (%) | | P-value | P-value ± SE after Montecarlo permutation |
| --- | --- | --- | --- | --- | --- | --- | --- |
| Study design | 11 | 0.03 ± 0.09 | -13 | | 0.729 | | 0.683 ± 0.014 |
| Male gender, RR | 10 | -0.10 ± 0.44 | -16 | | 0.819 | | 0.826 ± 0.012 |
| Age (years), MD | 10 | 0.02 ± 0.02 | -1 | | 0.431 | | 0.450 ± 0.016 |
| PDAC or CP, RR | 7 | 0.65 ± 0.36 | 25 | | 0.180 | | 0.202 ± 0.013 |
| “Soft pancreas”, RR | 7 | 0.16 ± 0.39 | -20 | | 0.692 | | 0.712 ± 0.014 |
| Use of somatostatine analogues, RR | 5 | -0.15 ± 0.43 | -31 | | 0.747 | | 0.729 ± 0.014 |
| Wirsung not dilated, RR | 6 | -0.34 ± 0.64 | -23 | | 0.617 | | 0.625 ± 0.015 |
| MINORS score | 11 | 0.01 ± 0.01 | -1 | | 0.449 | | 0.462 ± 0.019 |
| Type of BA (c-BA vs. m-BA) | 11 | 0.01 ± 0.09 | -17 | | 0.990 | | 0.987 ± 0.003 |
| Type of DtoM (CW-DtoM vs. Ka-DtoM) | 11 | -0.02 ± 0.09 | -16 | | 0.758 | | 0.759 ± 0.013 |
| Study Origin (Western vs. Eastern) | 10 | 0.17 ± 0.09 | 32 | | 0.081 | | 0.082 ± 0.009 |

**Legend**: SE=Standard Error; = Blumgart Anastomosis; CW-DtoM= Cattel Warren Duct to mucosa anastomosis; Ka-DtoM= Kakita Duct to mucosa anastomosis; PDAC= Pancreatic ductal adenocarcinoma; CP= chronic pancreatitis; RR= risk ratio; WMD= Wheigted Mean Mifference; R^2^= Relative reduction in between-study variance: the value indicates the proportion of between study variance explained by covariate; RR= Risk Ratio; MD=Mean Difference; BMI= Body Mass Index; PDAC = Pancreatic Ductal AdenoCarcinoma; CP=Chronic Pancreatitis; PP=Pylorus Preserving Pancreaticoduodenectomy ; *= insufficient observation to perform Monte Carlo permutation

**Supplementary Table 4. Results of univariate meta-regression analysis for post-pancreatectomy hemorrhage**

| Covariates | Number of studies | Beta coefficient ± SE | | Adjusted R^2^ (%) | | P-value | P-value ± SE after Montecarlo permutation |
| --- | --- | --- | --- | --- | --- | --- | --- |
| Study design | 11 | 0.02 ± 0.03 | -12 | | 0.503 | | 0.467 ± 0.026 |
| Male gender, RR | 11 | 0.02 ± 0.13 | -26 | | 0.870 | | 0.865 ± 0.011 |
| Age (years), MD | 10 | 0.01 ± 0.01 | -13 | | 0.466 | | 0.431 ± 0.016 |
| PDAC or CP, RR | 9 | 0.05 ± 0.10 | -50 | | 0.607 | | 0.622 ± 0.015 |
| “Soft pancreas”, RR | 8 | 0.10 ± 0.12 | -4 | | 0.445 | | 0.414 ± 0.016 |
| Use of somatostatine analogues, RR | 5 | -0.15 ± 0.07 | 72 | | 0.144 | | 0.170 ± 0.012 |
| Wirsung not dilated, RR | 5 | -0.18 ± 0.09 | 100 | | 0.098 | | 0.048 ± 0.007 |
| MINORS score | 11 | -0.01 ± 0.01 | -11 | | 0.413 | | 0.392 ± 0.021 |
| Type of BA (c-BA vs. m-BA) | 10 | 0.05 ± 0.02 | 76 | | 0.052 | | 0.060 ± 0.023 |
| Type of DtoM (CW-DtoM vs. Ka-DtoM) | 10 | 0.05 ± 0.03 | 0 | | 0.133 | | 0.170 ± 0.038 |
| Study Origin (Western vs. Eastern) | 10 | 0.07 ± 0.04 | 50 | | 0.109 | | 0.150 ± 0.035 |

**Legend**: SE=Standard Error; = Blumgart Anastomosis; CW-DtoM= Cattel Warren Duct to mucosa anastomosis; Ka-DtoM= Kakita Duct to mucosa anastomosis; PDAC= Pancreatic ductal adenocarcinoma; CP= chronic pancreatitis; RR= risk ratio; WMD= Wheigted Mean Mifference; R^2^= Relative reduction in between-study variance: the value indicates the proportion of between study variance explained by covariate; RR= Risk Ratio; MD=Mean Difference; BMI= Body Mass Index; PDAC = Pancreatic Ductal AdenoCarcinoma; CP=Chronic Pancreatitis; PP=Pylorus Preserving Pancreaticoduodenectomy ; *= insufficient observation to perform Monte Carlo permutation

**Supplementary Table 5. Results of univariate meta-regression analysis for delayed gastric emptying**

| Covariates | Number of studies | Beta coefficient ± SE | | Adjusted R^2^ (%) | | P value | P value ± SE after Montecarlo permutation |
| --- | --- | --- | --- | --- | --- | --- | --- |
| Study design | 8 | 0.02 ± 0.07 | -14 | | 0.749 | | 0.780 ± 0.041 |
| Male gender, RR | 8 | 0.33 ± 0.12 | 99 | | 0.080 | | 0.080 ± 0.027 |
| Age (years), MD | 8 | -0.01 ± 0.01 | -45 | | 0.963 | | 0.970 ± 0.017 |
| PDAC or CP, RR | 7 | 0.08 ± 0.12 | 0 | | 0.552 | | 0.490 ± 0.050 |
| “Soft pancreas”, RR | 7 | 0.42 ± 0.27 | 80 | | 0.167 | | 0.220 ± 0.041 |
| Use of somatostatin analogs, RR | 3 | -0.16 ± 0.23 | 0 | | 0.611 | | 0.800 ± 0.040 |
| Wirsung not dilated, RR | 5 | -0.39 ± 0.14 | 100 | | 0.068 | | **0.018 ± 0.001** |
| MINORS score | 8 | 0.01 ± 0.02 | 11 | | 0.354 | | 0.330 ± 0.047 |
| Type of BA (c-BA vs. m-BA) | 8 | 0.07 ± 0.04 | 44 | | 0.096 | | 0.080 ± 0.027 |
| Type of DtoM (CW-DtoM vs. Ka-DtoM) | 8 | -0.03 ± 0.04 | -22 | | 0.527 | | 0.560 ± 0.005 |
| Study Origin (Western vs. Eastern) | 8 | 0.05 ± 0.10 | -12 | | 0.591 | | 0.680 ± 0.046 |

**Legend**: SE=Standard Error; = Blumgart Anastomosis; CW-DtoM= Cattel Warren Duct to mucosa anastomosis; Ka-DtoM= Kakita Duct to mucosa anastomosis; PDAC= Pancreatic ductal adenocarcinoma; CP= chronic pancreatitis; RR= risk ratio; WMD= Wheigted Mean Mifference; R^2^= Relative reduction in between-study variance: the value indicates the proportion of between study variance explained by covariate; RR= Risk Ratio; MD=Mean Difference; BMI= Body Mass Index; PDAC = Pancreatic Ductal AdenoCarcinoma; CP=Chronic Pancreatitis; PP=Pylorus Preserving Pancreaticoduodenectomy ; *= insufficient observation to perform Monte Carlo permutation

**Supplementary Table 6. Results of univariate meta-regression analysis for length of stay**

| Covariates | Number of studies | Beta coefficient ± SE | | Adjusted R^2^ (%) | | P value | P value ± SE after Montecarlo permutation |
| --- | --- | --- | --- | --- | --- | --- | --- |
| Male gender, RR | 12 | 0.10 ± 13.78 | 28 | | 0.180 | | 0.182 ± 0.012 |
| Age (years), MD | 12 | -0.39 ± 0.96 | -13 | | 0.691 | | 0.715 ± 0.014 |
| PDAC or CP, RR | 9 | -4.45 ± 10.94 | -22 | | 0.698 | | 0.699 ± 0.015 |
| “Soft pancreas”, RR | 8 | 10.97 ± 12.50 | -6 | | 0.414 | | 0.426 ± 0.016 |
| Use of somatostatine analogues, RR | 5 | 0.9 ± 14.52 | -45 | | 0.955 | | 0.977 ± 0.005 |
| Wirsung not dilated, RR | 6 | -41.58 ± 9.67 | 100 | | 0.023 | | 0.011 ± 0.003 |
| MINORS score | 12 | 0.29 ± 0.59 | -9 | | 0.635 | | 0.649 ± 0.015 |
| Type of BA (c-BA vs. m-BA) | 12 | 1.19 ± 3.58 | -11 | | 0.748 | | 0.720 ± 0.014 |
| Type of DtoM (CW-DtoM vs. Ka-DtoM) | 12 | 0.98 ± 3.51 | -13 | | 0.787 | | 0.775 ± 0.013 |
| Study Origin (Western vs. Eastern) | 12 | -1.11 ± 4.55 | -12 | | 0.812 | | 0.754 ± 0.014 |

**Legend**: SE=Standard Error; = Blumgart Anastomosis; CW-DtoM= Cattel Warren Duct to mucosa anastomosis; Ka-DtoM= Kakita Duct to mucosa anastomosis; PDAC= Pancreatic ductal adenocarcinoma; CP= chronic pancreatitis; RR= risk ratio; WMD= Wheigted Mean Mifference; R^2^= Relative reduction in between-study variance: the value indicates the proportion of between study variance explained by covariate; RR= Risk Ratio; MD=Mean Difference; BMI= Body Mass Index; PDAC = Pancreatic Ductal AdenoCarcinoma; CP=Chronic Pancreatitis; PP=Pylorus Preserving Pancreaticoduodenectomy ; *= insufficient observation to perform Monte Carlo permutation
