# Supplementary material for: Blumgart Anastomosis After Pancreaticoduodenectomy. A Comprehensive Systematic Review, Meta-Analysis, and Meta-Regression
Source: World J Surg. 2021 Mar 15;45(6):1929–39. doi: 10.1007/s00268-021-06039-x (PMC8093149; doi:10.1007/s00268-021-06039-x)
Supplement: Supplementary file 2 — Supplementary file2 (DOCX 43069 KB) [file 268_2021_6039_MOESM2_ESM.docx]

**Supplementary Figures**

**Supplementary Figure 1A:** Funnel plot of Clinical Relevant Post-operative pancreatic fistula (CR-POPF)


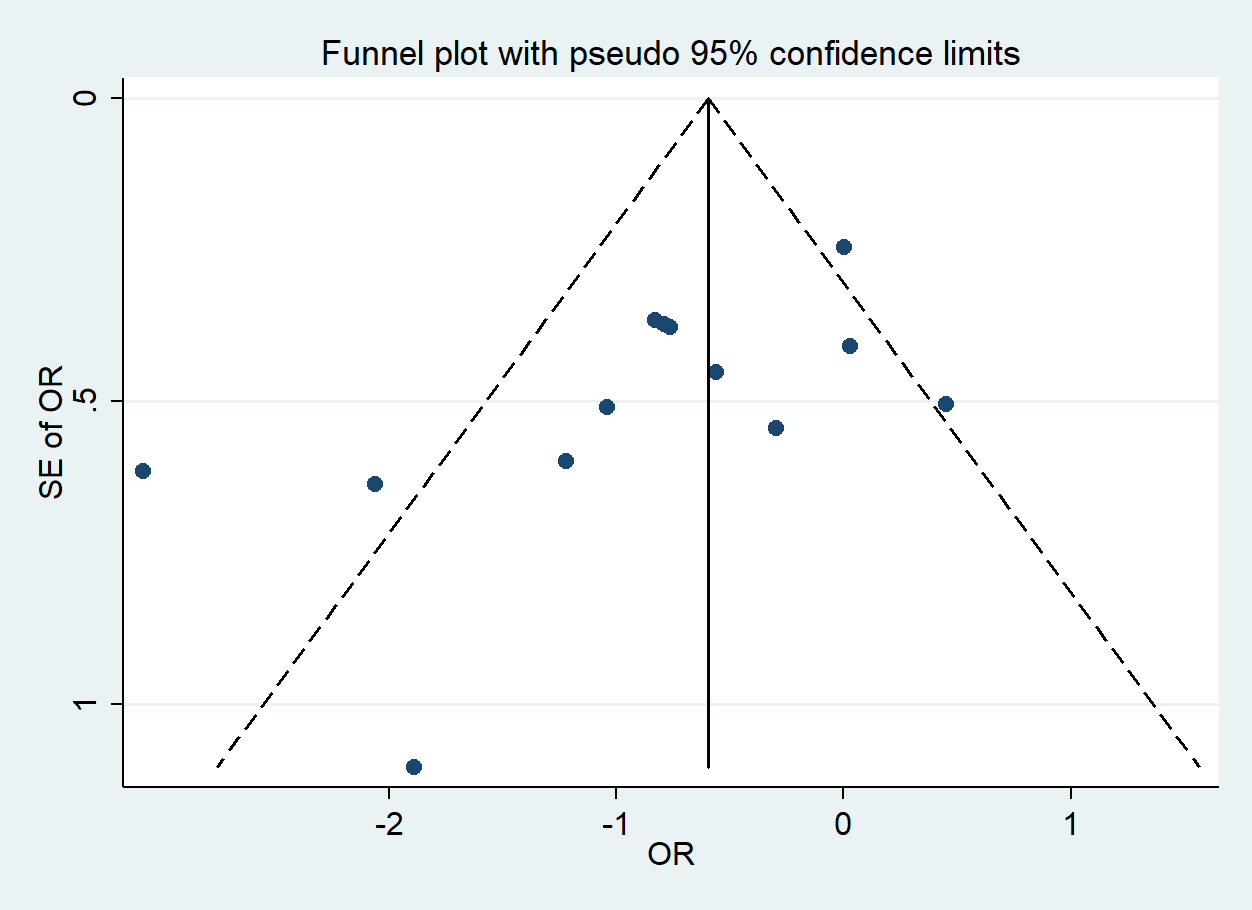


**Legend:** Each blue point represents the included studies. Black dashed sloping lines represent the confidence interval at 95 %. The solid black line indicates the random-effects summary estimate using inverse variance weighting.

**Supplementary Figure 1B:** Funnel plot of Clinical Relevant Post-operative pancreatic fistula based on Odds Ratios values of multivariate analysis (CR-POPF)

**
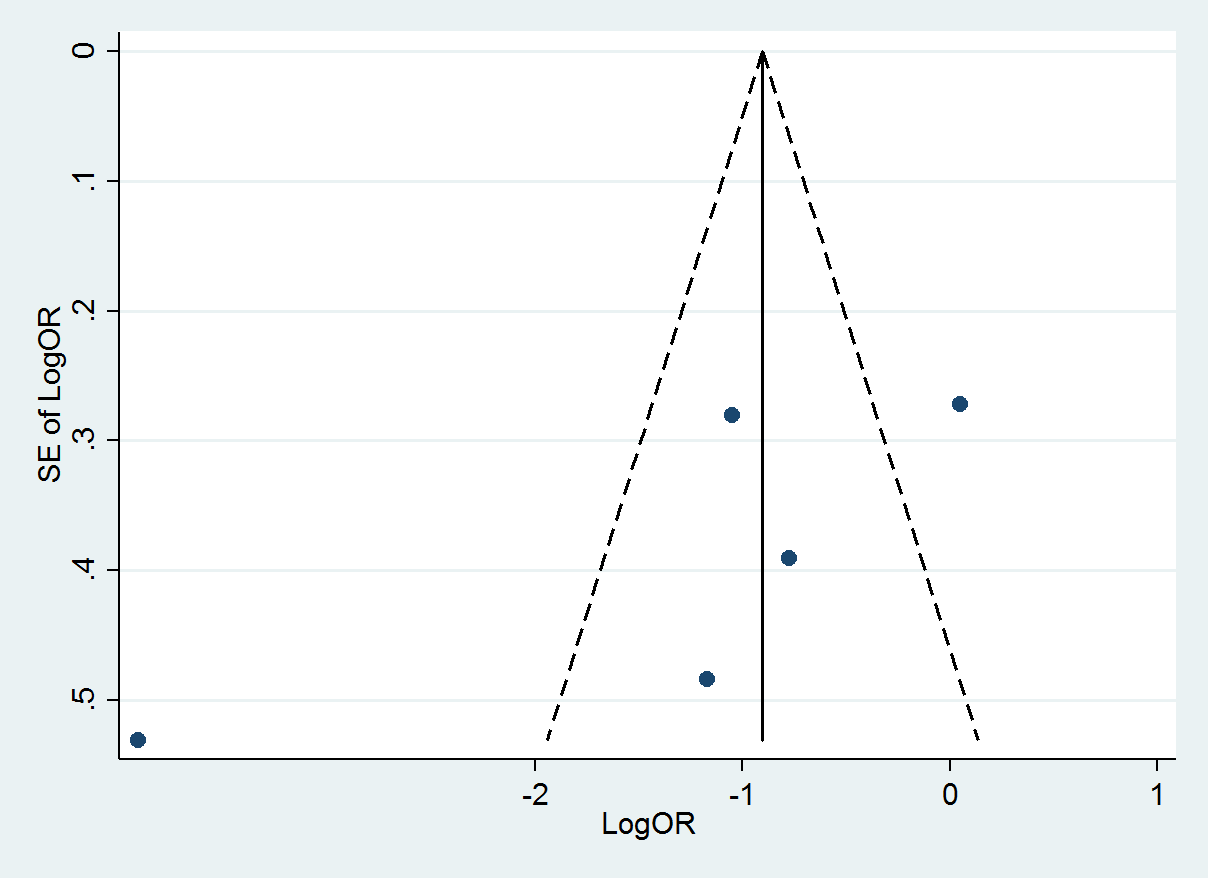
**

**Legend:** Each blue point represents the included studies. Black dashed sloping lines represent the confidence interval at 95 %. The solid black line indicates the random-effects summary estimate using inverse variance weighting.

**Supplementary Figure 1C:** Funnel plot of postoperative mortality


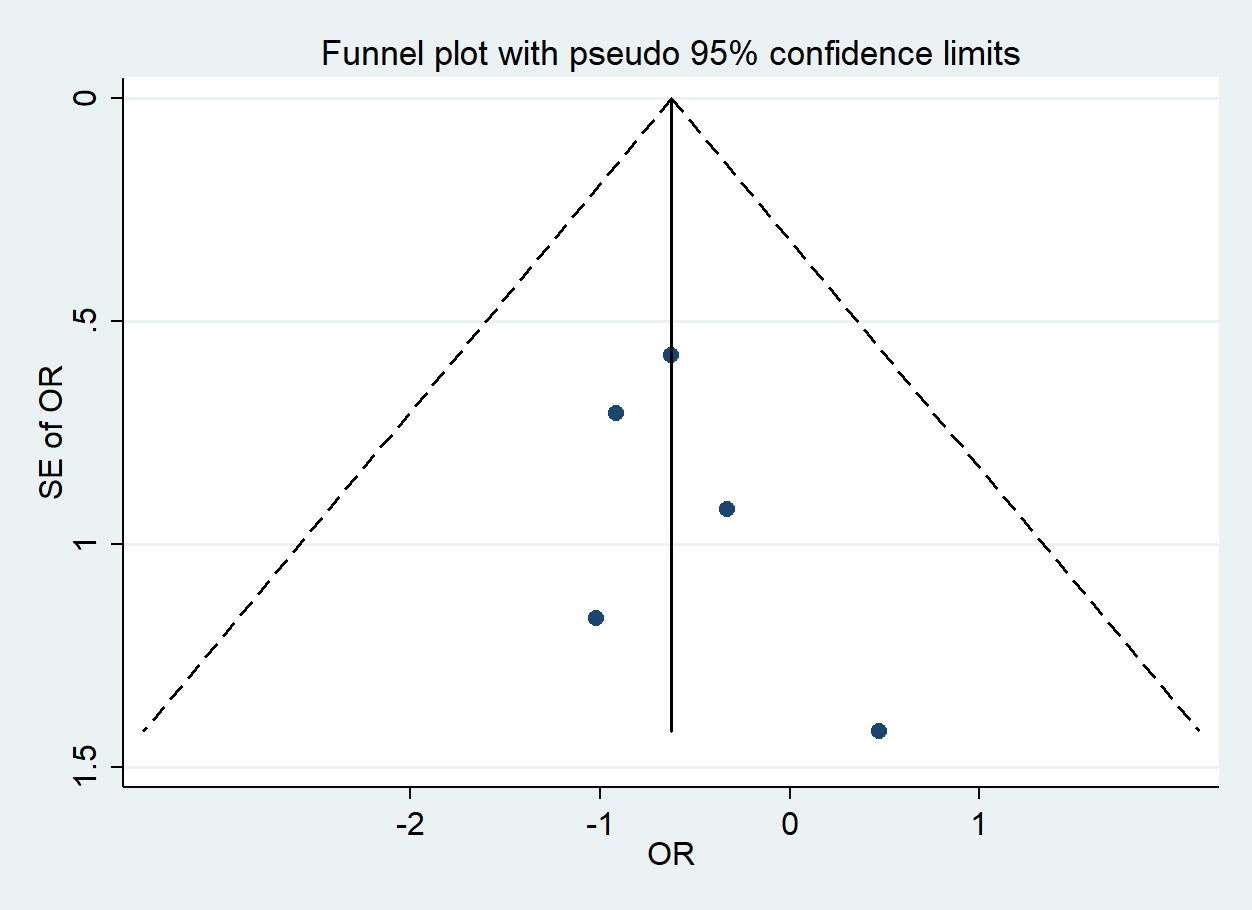


**Legend:** Each blue point represents the included studies. Black dashed sloping lines represent the confidence interval at 95 %. The solid black line indicates the random-effects summary estimate using inverse variance weighting.

**Supplementary Figure 1D:** Funnel plot of postoperative morbidity

**
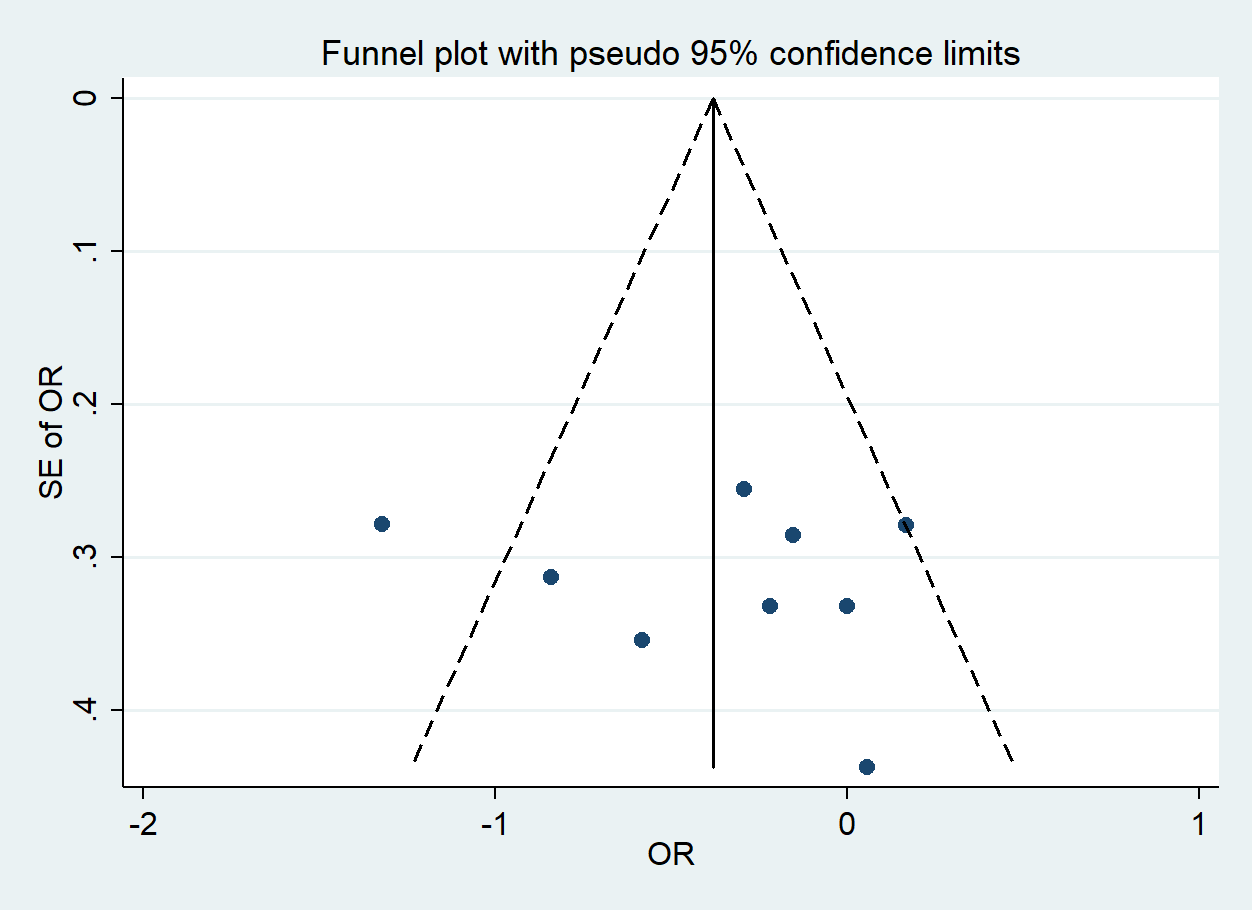
**

**Legend:** Each blue point represents the included studies. Black dashed sloping lines represent the confidence interval at 95 %. The solid black line indicates the random-effects summary estimate using inverse variance weighting.

**Supplementary Figure 1E:** Funnel plot of post-pancreatectomy hemorrhage (PPH)


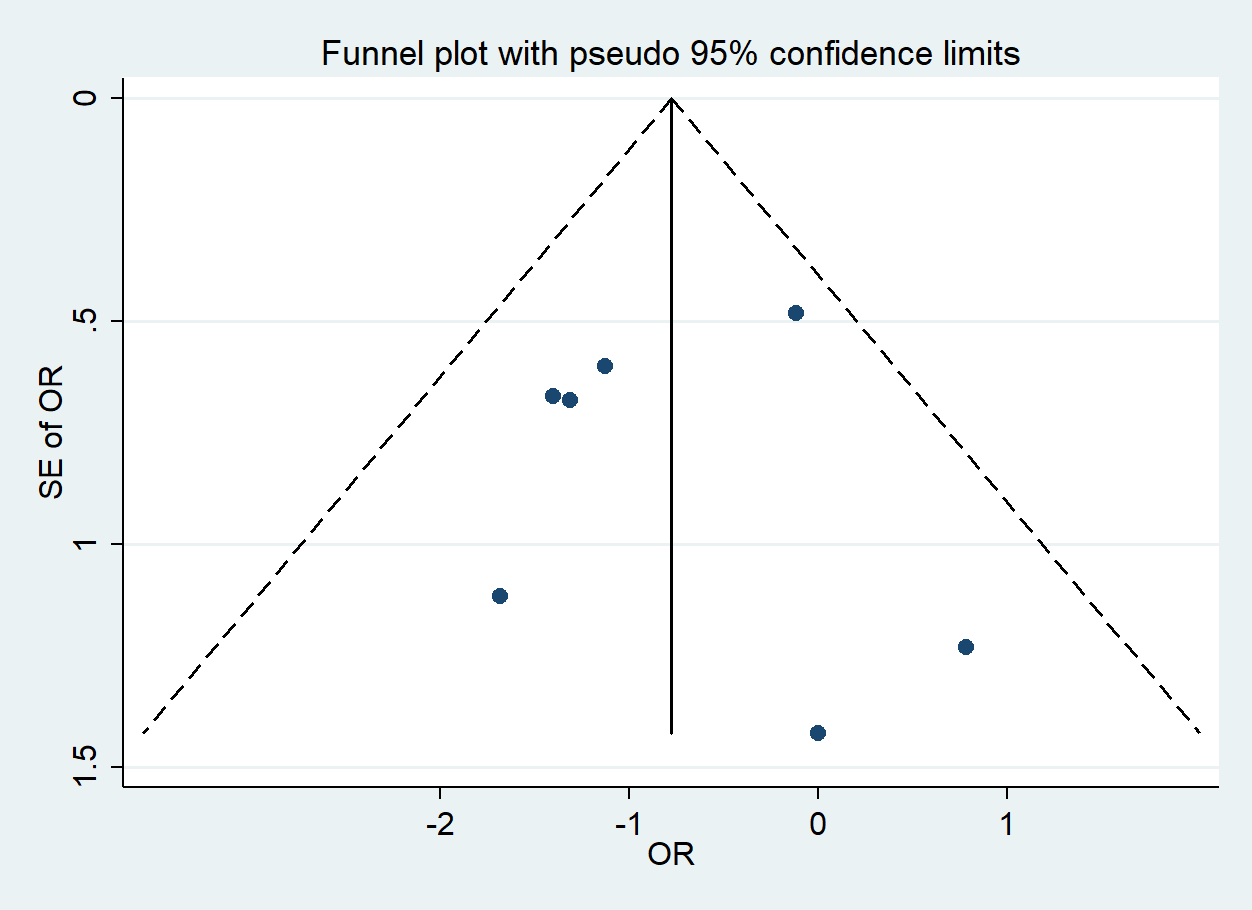


**Legend:** Each blue point represents the included studies. Black dashed sloping lines represent the confidence interval at 95 %. The solid black line indicates the random-effects summary estimate using inverse variance weighting.

**Supplementary Figure 1F:** Funnel plot of delayed gastric emptying (DGE)


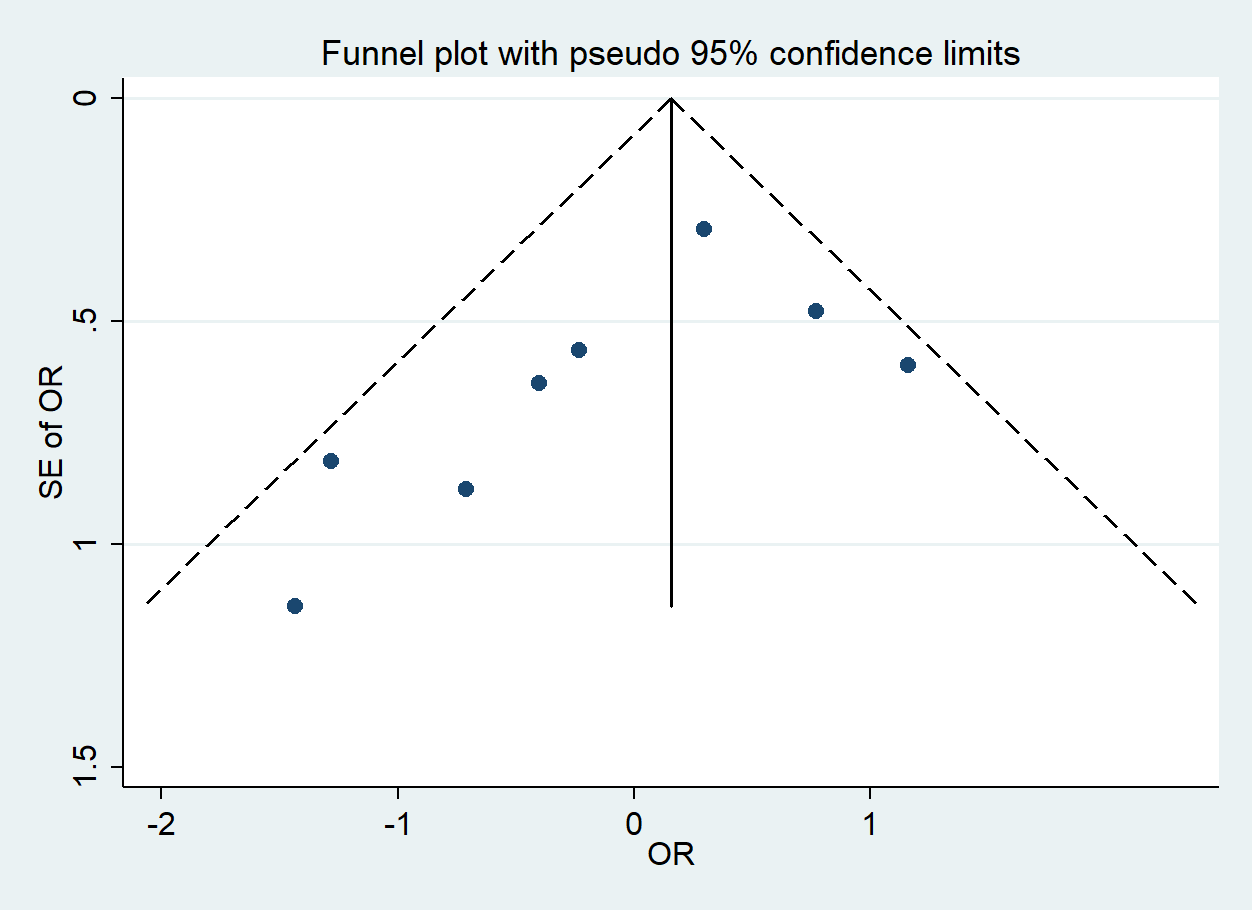


**Legend:** Each blue point represents the included studies. Black dashed sloping lines represent the confidence interval at 95 %. The solid black line indicates the random-effects summary estimate using inverse variance weighting.

**Supplementary Figure 1G:** Funnel plot of reoperation rate


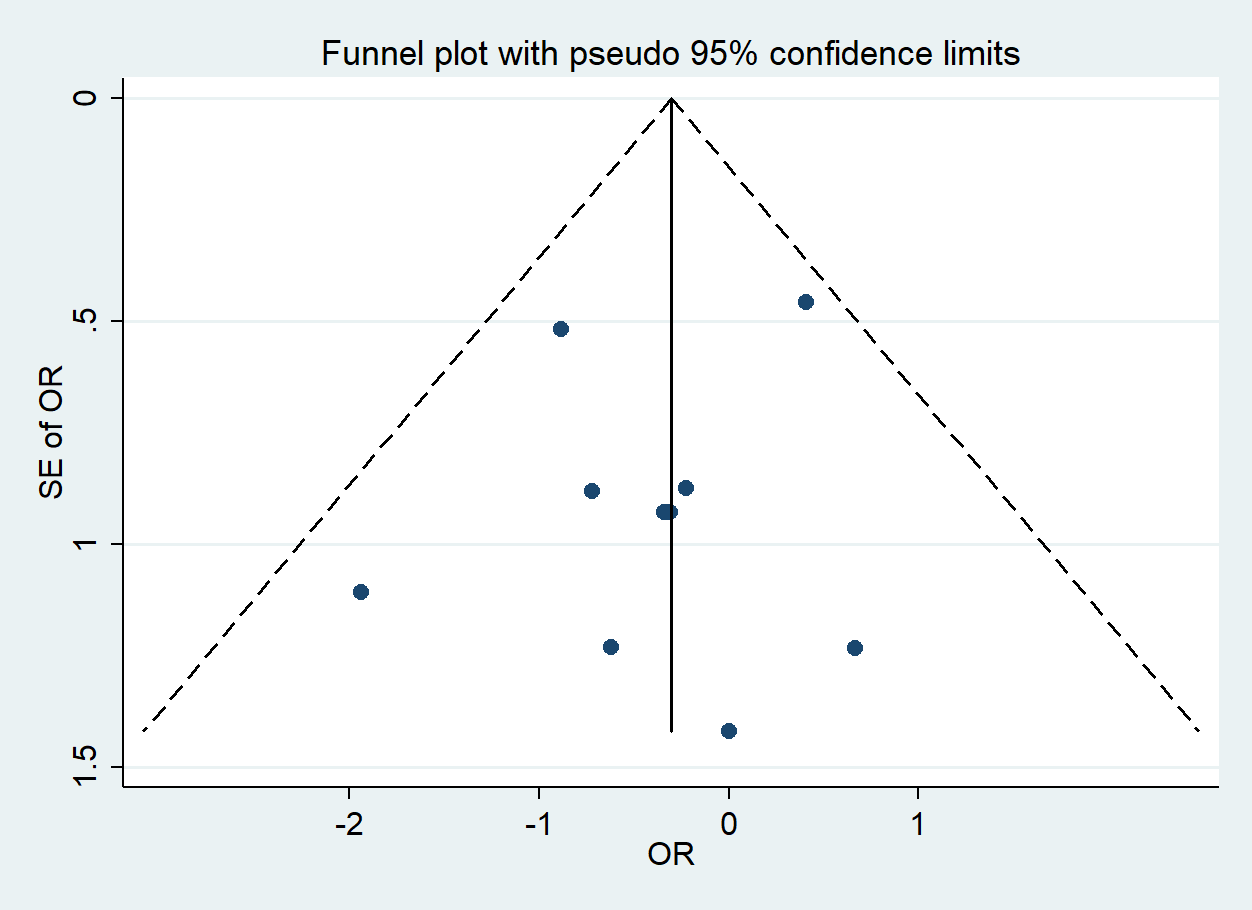


**Legend:** Each blue point represents the included studies. Black dashed sloping lines represent the confidence interval at 95 %. The solid black line indicates the random-effects summary estimate using inverse variance weighting.

**Supplementary Figure 2 panel A:** Forest plot of mortality rate


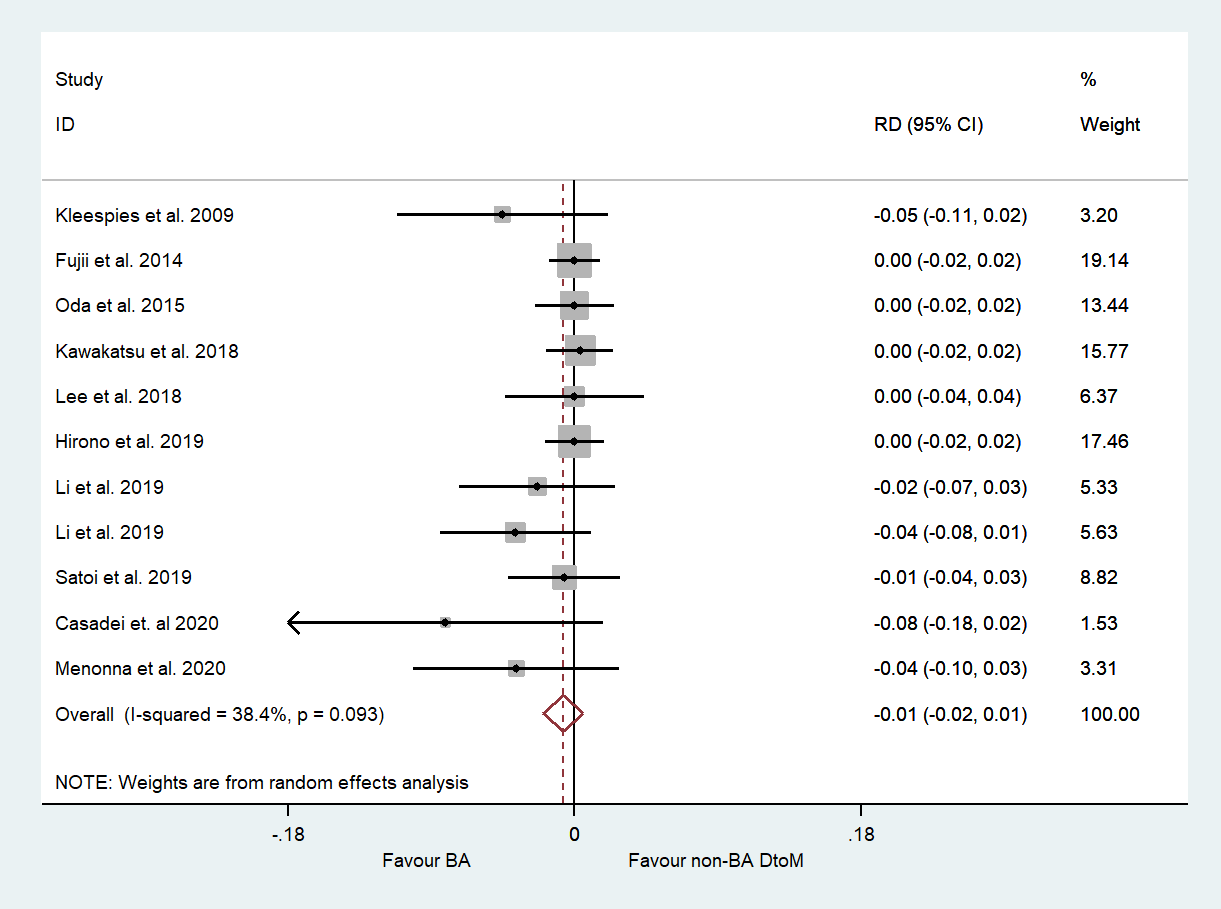


**Legend:** RD=Risk difference; 95%CI: the 95% confidence interval; I-squared: between-study heterogeneity according to Higgins’s test; p= p-value referred to Q Cochran test; gray square: risk difference of each study; Size of square: the weight of each study in the analysis; Solid black line: the 95% confidence interval for each study; Red diamond: the pooled risk difference

**Supplementary Figure 2 panel B:** Forest plot of morbidity rate


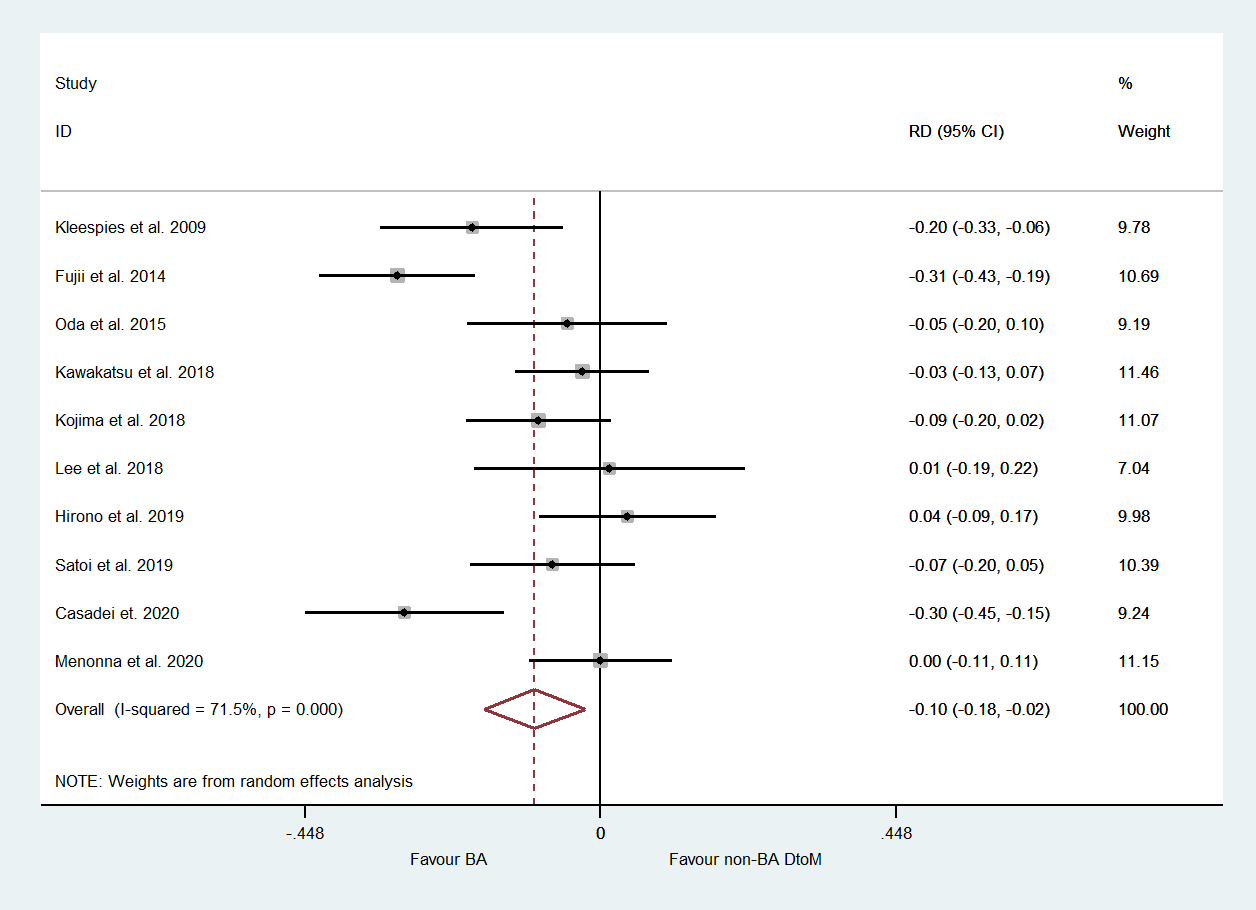


**Legend:** RD=Risk difference; 95%CI: the 95% confidence interval; I-squared: between-study heterogeneity according to Higgins’s test; p= p-value referred to Q Cochran test; gray square: risk difference of each study; Size of square: the weight of each study in the analysis; Solid black line: the 95% confidence interval for each study; Red diamond: the pooled risk difference

**Supplementary Figure 2 panel C:** Forest plot of post-pancreatectomy hemorrhage (PPH) rate


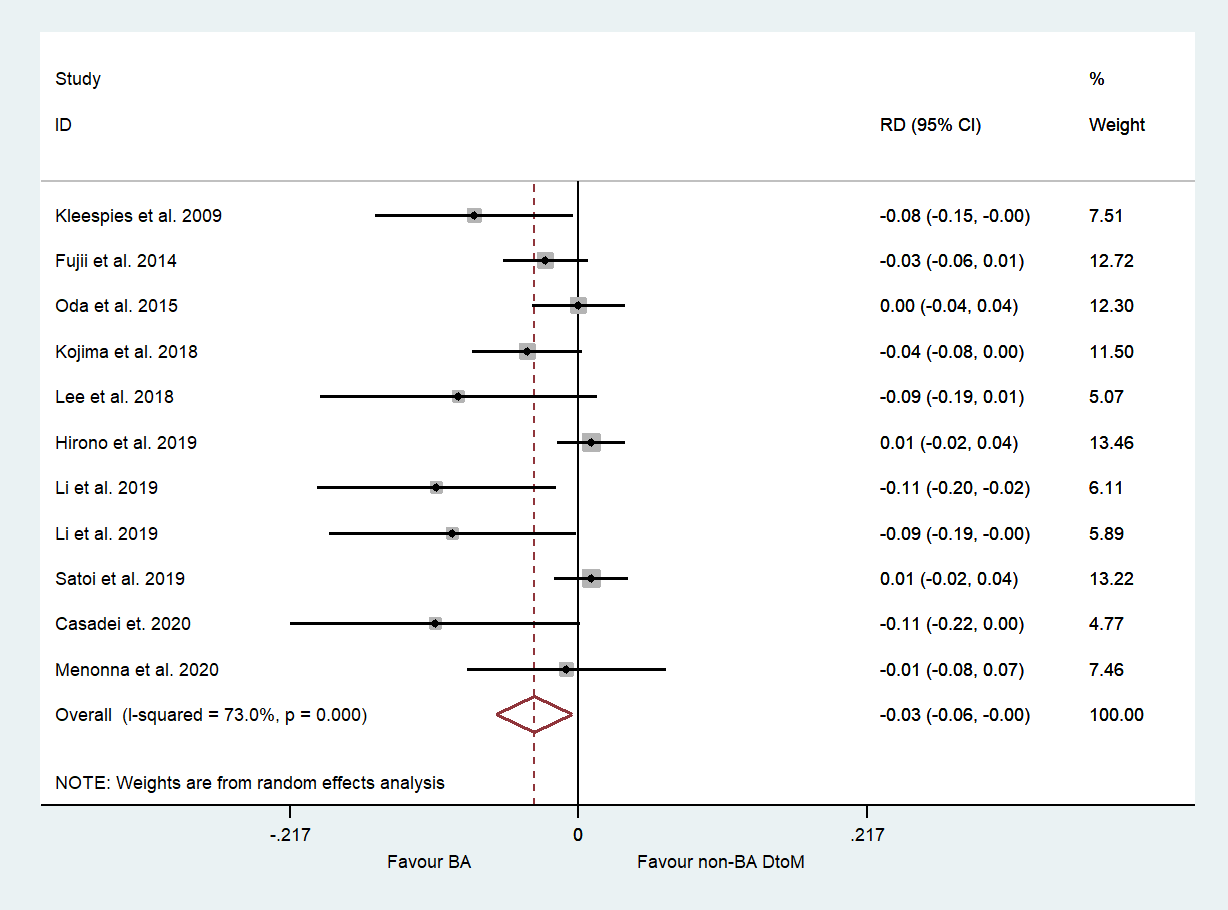


**Legend:** RD=Risk difference; 95%CI: the 95% confidence interval; I-squared: between-study heterogeneity according to Higgins’s test; p= p-value referred to Q Cochran test; gray square: risk difference of each study; Size of square: the weight of each study in the analysis; Solid black line: the 95% confidence interval for each study; Red diamond: the pooled risk difference

**Supplementary Figure 2 panel D:** Forest plot of delayed gastric emptying (DGE) rate


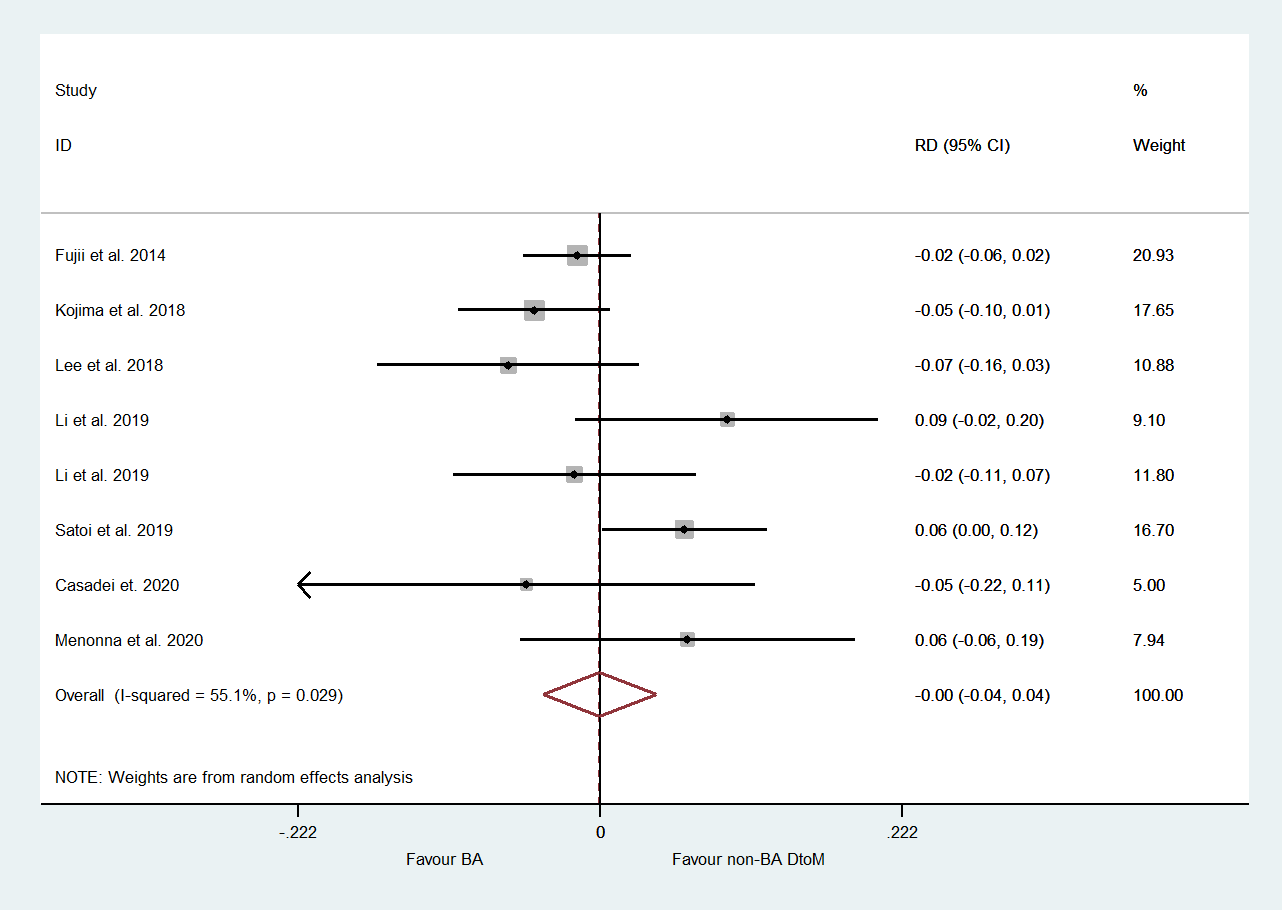


**Legend:** RD=Risk difference; 95%CI: the 95% confidence interval; I-squared: between-study heterogeneity according to Higgins’s test; p= p-value referred to Q Cochran test; gray square: risk difference of each study; Size of square: the weight of each study in the analysis; Solid black line: the 95% confidence interval for each study; Red diamond: the pooled risk difference

**Supplementary Figure 2 panel E:** Forest plot of reoperation rate


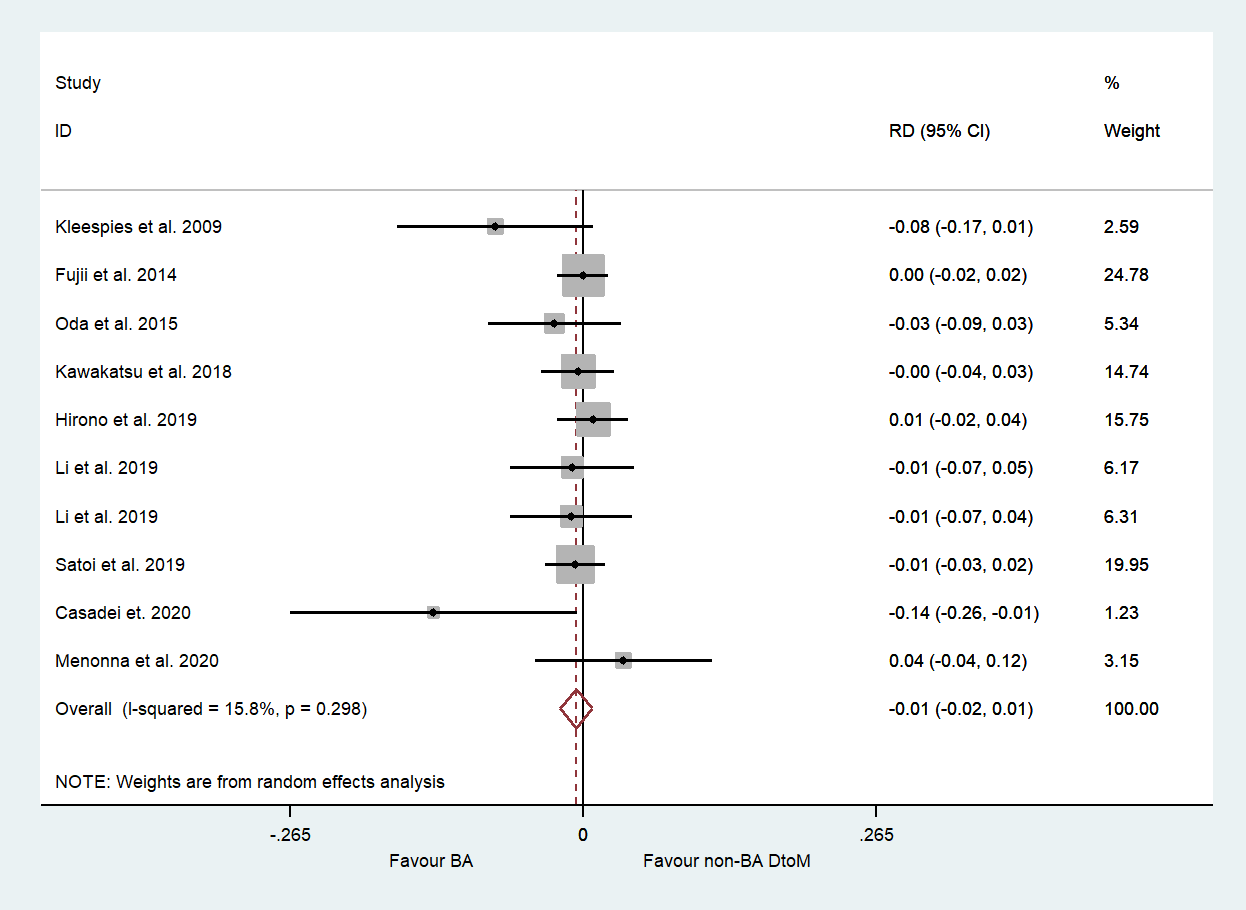


**Legend:** RD=Risk difference; 95%CI: the 95% confidence interval; I-squared: between-study heterogeneity according to Higgins’s test; p= p-value referred to Q Cochran test; gray square: risk difference of each study; Size of square: the weight of each study in the analysis; Solid black line: the 95% confidence interval for each study; Red diamond: the pooled risk difference

**Supplementary Figure 2 panel F:** Forest plot of length of post-operative stay


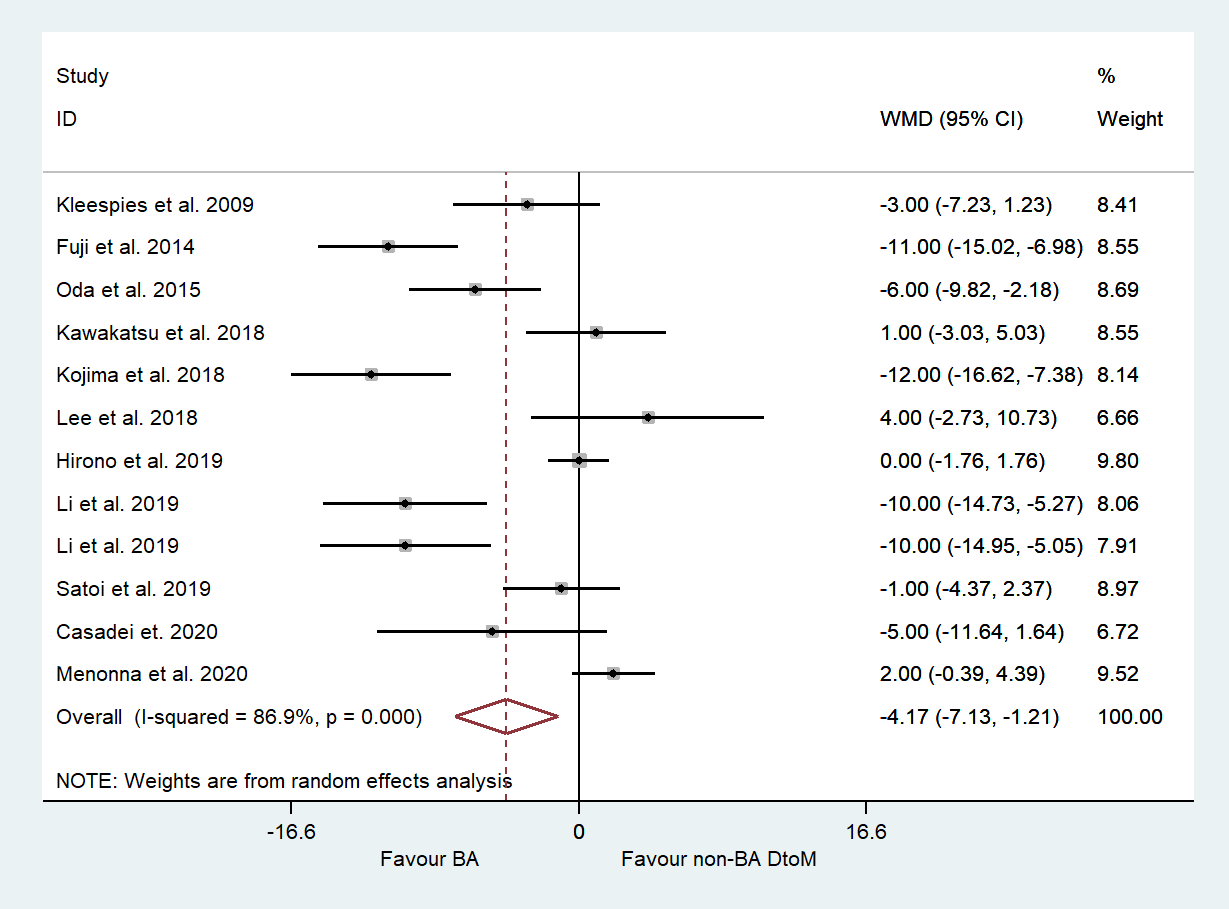


**Legend:** WMD=weighted mean difference; 95%CI: the 95% confidence interval; I-squared: between-study heterogeneity according to Higgins’s test; p= p-value referred to Q Cochran test; gray square: risk difference of each study; Size of square: the weight of each study in the analysis; Solid black line: the 95% confidence interval for each study; Red diamond: the pooled risk difference
